# Application of Dense Neural Networks for Detection of Atrial Fibrillation and Ranking of Augmented ECG Feature Set

**DOI:** 10.3390/s21206848

**Published:** 2021-10-15

**Authors:** Vessela Krasteva, Ivaylo Christov, Stefan Naydenov, Todor Stoyanov, Irena Jekova

**Affiliations:** 1 Institute of Biophysics and Biomedical Engineering, Bulgarian Academy of Sciences, Acad. G. Bonchev Str. Bl 105, 1113 Sofia, Bulgaria; vessika@biomed.bas.bg (V.K.); ivaylo.christov@biomed.bas.bg (I.C.); todor@biomed.bas.bg (T.S.); 2 Department of Internal Diseases “Prof. St. Kirkovich”, Medical University of Sofia, 1431 Sofia, Bulgaria; snaydenov@gmail.com

**Keywords:** artificial neural network, ECG, PhysioNet Computing in Cardiology Challenge 2017 database, atrial fibrillation, arrhythmia detection, noise detection, feature importance, neuron weights, optimization, deep learning

## Abstract

Considering the significant burden to patients and healthcare systems globally related to atrial fibrillation (AF) complications, the early AF diagnosis is of crucial importance. In the view of prominent perspectives for fast and accurate point-of-care arrhythmia detection, our study optimizes an artificial neural network (NN) classifier and ranks the importance of enhanced 137 diagnostic ECG features computed from time and frequency ECG signal representations of short single-lead strips available in 2017 Physionet/CinC Challenge database. Based on hyperparameters’ grid search of densely connected NN layers, we derive the optimal topology with three layers and 128, 32, 4 neurons per layer (DenseNet-3@128-32-4), which presents maximal F1-scores for classification of Normal rhythms (0.883, 5076 strips), AF (0.825, 758 strips), Other rhythms (0.705, 2415 strips), Noise (0.618, 279 strips) and total F1 relevant to the CinC Challenge of 0.804, derived by five-fold cross-validation. DenseNet-3@128-32-4 performs equally well with 137 to 32 features and presents tolerable reduction by about 0.03 to 0.06 points for limited input sets, including 8 and 16 features, respectively. The feature reduction is linked to effective application of a comprehensive method for computation of the feature map importance based on the weights of the activated neurons through the total path from input to specific output in DenseNet. The detailed analysis of 20 top-ranked ECG features with greatest importance to the detection of each rhythm and overall of all rhythms reveals DenseNet decision-making process, noticeably corresponding to the cardiologists’ diagnostic point of view.

## 1. Introduction

Atrial fibrillation (AF) is the most common sustained cardiac arrhythmia in adults: the lifelong risk is ~25%, i.e., one in four will have at least one episode of AF [1]. This rhythm disorder is associated with substantial morbidity and mortality, mostly due to ischemic strokes: ~20% of the non-anticoagulated AF patients will develop a stroke, and one of three to five strokes is due to AF. The significant burden to patients and healthcare systems globally, related to AF complications, necessitates the early diagnosing of this arrhythmia [1,2,3,4].

Confirmation of AF requires rhythm documentation with an electrocardiogram (ECG), showing heart rhythm with no discernible P-waves and irregular RR intervals [1,5]. However, in the real clinical practice, many patients with recurrent episodes of paroxysmal AF are often in normal sinus rhythm at the time of clinical visits [1,6]. On the other hand, short and asymptomatic AF episodes possess similar stoke potential as the longer ones, and it is not uncommon AF patients to present first with an ischemic stroke [1,2,3]. Since standard 12-lead ECG strips during clinical visits are unreliable to confirm or reject recurrent episodes of AF, clinicians count a lot on ECG monitoring techniques like 24–72 h Holter-ECG monitors and loop recorders, tracing patient’s rhythm for days to several weeks [1,6,7]. Today, these methods have become routine to detect paroxysmal, self-limited rhythm and conductance disorders often not detected by short-term ECG strips [1,5,7]. However, they turn out to not be the perfect solution for arrhythmia diagnosis for many patients [5,6,7]. Most of the ECG monitoring devices are single- to three-lead and may not reliably depict important components for the correct diagnosis of the record, particularly the low-amplitude ‘P’-waves of normal atrial depolarisation in sinus rhythm from the ‘saw-like’ waves of atrial excitation in atrial flutter or the fine deviations of the isoelectric line (’f’-waves) in AF [5,6,7,8]. Differentiation between atrial fibrillation and flutter may become even more difficult if the unidentifiable waves of atrial excitation are combined with alternating atrio–ventricular conductance. On the other hand, no detected irregular rhythm means AF [5,6,7,8,9]. In addition, Holter ECG monitoring is performed during patients’ daily activities, and the signal is highly affected by noise and artifacts: noise could be a matter of major concern because its presence may result in false diagnoses [8,9,10]. The main sources of noise are patient movements, causing baseline wander noise, electromyographic noise and electrode motion, powerline or electronic device interference, signal processing, medical equipment, etc. [7,8,9,10]. Therefore, real-time lead quality monitoring is important for providing a diagnostically reliable data, and systems have been developed to trigger the start of the recording at potentially the best quality [11].

The AF detection from the ECG signal generally relies on analysis of the atrial activity for presence of multiple P-waves [12,13] or absence of P-waves [14,15]; analysis of the ventricular response time and particularly the median absolute deviation of RR intervals [16], their irregularity [17] and sample entropy [18]; and joined analysis of the independent data from the atrial and ventricular activity [19,20]. RR interval irregularity is recognized in noise-robust AF detectors with high sensitivity and specificity, whereas those with highest positive predictive value rely on the combined analysis of RR intervals and atrial activity [21].

To motivate research that overcomes the traditional difficulties encountered by conventional ECG monitors for AF, in noisy conditions and various arrhythmias, the PhysioNet Computing in Cardiology (CinC) Challenge 2017 has recently focused on the AF detection from short-time single-lead ECG strips [22], involving 75 international competitors. Selected papers have been published in a dedicated Special Issue ‘Focus on detection of arrhythmia and noise from cardiovascular data’ in *Physiological Measurement* [23]. All included papers can be considered as a truthful basis of related works to this study; therefore, a detailed list of embedded classifiers and input features is further reviewed:

- Support vector machine (SVM) classifiers are input with 47 features from the statistical and morphological rhythm representation [24]; 33 features expressive to the signal power, spectrum, entropy, RR intervals and P-waves [25]; and 61 features from the time-frequency ECG representation, both average and variability of RR intervals, and the average beat morphology [26];
- Linear and quadratic discriminant classifiers are input with a set of 122 RR-interval features from their time domain, frequency domain and distribution (histogram) representations [27] and 44 features measured by heart rate variability (HRV) analysis, average beat morphology analysis, and atrial activity analysis focused on the P-wave amplitude in the average beat and f-waves amplitude in TQ intervals [28];
- Decision tree classifiers are input with 30 multi-level features, including measures of AF, morphology, RR intervals and similarity between beats [29]; morphological coefficients and HRV features calculated by ECG waveform fitting with a piecewise linear spline [30]; 400 hand-crafted features, reflecting the complex physiology of cardiac arrhythmias visible in single-channel ECG [31]; and 74 features of the R-peak amplitude, RR-interval statistics, PQRST statistics, ECG signal irregularity, entropy, noise content and four additional sparse coding features [32];
- A multi-layer binary classification architecture is input with subsets of 77, 66 and 19 features selected from 188 dimensional feature pool containing time, frequency, morphological and statistical domain ECG features [33];
- A multi-stage classifier, combining SVM, decision tree and threshold-based rules is quantifying both atrial and ventricular activity, estimated by local features (beat classification) and global features (rhythm, signal quality, similarity) [34].
- Advanced multi-stage classifiers, combining decision trees and neural networks (NNs) include: a tree gradient boosting model and a recurrent long, short-term memory (LSTM) network as a global classifier that uses 42 summary ECG features of the full record and a sequence classifier that works on a beat-by-beat basis using individual features of each cardiac cycle [35]; a bagged tree ensemble with 43 input features based on QRS detection and PQRS morphology connected in parallel to a convolutional neural network (CNN) and a shallow NN for analysis of raw filtered ECG signals (8× envelograms, 1× band-pass) [36]; a nine-layer CNN for segmentation of P, QRS and T waves, inter-beat segments, noise and arrhythmic beats, additionally augmented by hand-crafted features, thus supplying a set of 181 features to eXtreme Gradient Boosting trees to classify the heart rhythm [37]; a densely connected CNN applied on time–frequency ECG spectrograms (9 s, 15 s) and subsequent refined classification by AdaBoost-abstain classifier of 437 features, including signal quality, frequency content, RR-interval, ECG-based reconstructed phase space and Poincaré plots [38]; and ENCASE as an ensemble of multiple gradient-boosting decision trees with 590 input features, including deep features extracted with a deep neural network (DNN) from raw ECG and engineering features (statistical, signal, morphological and unsupervised) [39];
- DNN classifiers, including a quadratic NN input with a set of 122 RR-interval features from their time domain, frequency domain and distribution (histogram) representations [27]; a 21-layer 1D convolutional recurrent neural network (RhythmNet) containing 16 CNN layers for raw ECG feature extraction followed by three recurrent layers for processing of ECG records with varying lengths [40]; and a combination of one 1D CNN layer and a sequence of three LSTM layers for raw ECG feature extraction and classification of arrhythmia [41].

The interest to the AF detection problem is intensified during the last three years after the Challenge, showing an advanced progress in ECG signal processing by DNN. Some basic neural architectures are further disclosed as an overview of the enhanced ECG representation of local and global rhythm information in deep layers, including: hybrid attention-based DNN (HADLN), embedding residual network (ResNet) and bidirectional Bi-LSTM architectures with attention mechanism to improve the interpretability of the model [42]; beat–interval–texture convolutional neural network (BIT-CNN) for image analysis of 2D time–morphology representations of the heartbeats (electrocardiomatrix) [43]; MultiFusionNet for multiplicative fusion of two DNNs trained on different sources of knowledge, including raw ECG data and large feature set from the Poincaré plot, average beat, cross correlation, fiducial points (intervals and amplitudes), presence of P-waves and atypical ventricular morphologies [44]; and an end-to-end 1D CNN with 10 convolutional blocks, two fully-connected layers, and an output Softmax classifier that uses only raw ECG input with data length normalization [45].

While the latter studies have been focused on multi-rhythm classification involving AF, as defined in the PhysioNet CinC Challenge 2017 framework, there are several studies that solve the binary AF/Non-AF classification problem. They present: RSL_ANN as a fully connected artificial NN with repeated structuring and learning procedure for processing of 19 ECG features (morphological, f-waves and HRV) [46]; HAN-ECG as an interpretable bidirectional-recurrent NN, employing three attention mechanisms for a multi-resolution AF analysis [47]; convolutional and recurrent NNs for extraction of high level features from segments of RR intervals [48]; and fully CNN architectures for 2D matrix processing from short-term Fourier transform and wavelet transform of input ECG [49].

Furthermore, attempts to reveal the importance of the input feature map have been presented with explanatory focus to the decision mechanisms in different machine learning classifiers. For example, the feature importance is estimated by the Gini index in a random forest classifier [31]; ‘weight’ method for Extreme Gradient Boosting classifier [31]; the bagged decision tree ensemble [29]; Gain index for decision trees [37]; minimum redundancy, maximum relevance and minimum redundancy maximum relevance justified with a decision trees ensemble [32]; minimum redundancy maximum relevance, decision tree and SVM ranking methods [33]; and logistic regression, permutation testing, random forest and SHapley Additive exPlanations (SHAP) [50]. The latter has shown that each feature importance technique results in different feature rankings, depending on their characteristics and assumptions. Furthermore, we suggest that different features might have specific importance for each output class. To our knowledge, there is no study that has investigated the features’ class dependency ranking of AF and other rhythms within the AF detection framework with NNs.

This study aims to gain knowledge for the performance and importance of an extended set of 137 hand-crafted ECG features used in shallow dense NNs for classification of AF, normal rhythm, other arrhythmia and noise. We aim to derive comprehensive formula for quantification the importance of the feature map to produce high probability for specific class prediction, thus trying to light up the decision-making process by the internal neural interactions in the learnt and optimized hidden dense layers. The performances of all features and a reduced set ranked by their importance are compared to other published studies with hand-crafted ECG features.

## 2. Materials and Methods

### 2.1. ECG Database

We use the PhysioNet CinC Challenge 2017 database [22,51], which has been distributed to encourage the development of methods to classify heart arrhythmia from easily accessible ECG signal sources within a short timeframe. The publicly available dataset comprises 8528 ECG recordings (median duration 30 s, range 9–61 s) in a single lead derived between the fingertips of the two arms (lead I equivalent). Note that the ECG signals are representative to the recording conditions met by wearable ECG devices with a hand-held Ag/AgCl electrode–skin contact susceptible to movement artifacts. Therefore, ECG signals are band pass filtered by the native device in a narrow bandwidth 0.5–40 Hz, limited to ±5 mV dynamic range and 300 Hz sampling rate. The records are labelled by expert annotators in four rhythm classes, Normal sinus rhythms (class N), AF (class AF), Other arrhythmia (class O: all non-AF abnormal rhythms) and Noise (class X: too noisy to classify records), included in the publicly available V3 version of labelling (Table 1).

The total database (DB) is initially split into 5 non-overlapping parts in a stratified manner so each rhythm is equally distributed in all DB parts (Table 1). This is done to provide the input for 5-fold cross-validation, where 4 DB parts (80%) are used for training and 1 DB part (20%) is used for the validation. Note that we do not use the hidden test set from the Challenge, as it is inaccessible to the public.

### 2.2. ECG Features

We present an augmented set of 137 diagnostic ECG features that gathers the knowledge from 8 analysis techniques with an extended view to the time and frequency ECG signal representation, including:

1. Noise detection—1 feature;
2. HRV analysis—21 features;
3. Average beat morphology analysis—25 features;
4. Heartbeat classification and rhythm analysis—19 features;
5. Principal component analysis of PQRST and TQ-intervals—5 features;
6. P-wave analysis (time domain)—12 features;
7. TQ-segment analysis (time domain)—10 features;
8. TQ-segment analysis (frequency domain)—44 features.

Definitions of the features are disclosed in Table 2. More details can be found in the cited references, as well as in the free Matlab code for their computation distributed along the PhysioNet CinC Challenge 2017 competitors [52,53] and available at: [https://physionet.org/content/challenge-2017/1.0.0/sources/ivaylo-christov-204.zip] and [https://physionet.org/content/challenge-2017/1.0.0/sources/irena-jekova-204.zip], accessed on 1 September 2021.

### 2.3. Dense Neural Network Classifier

**Figure 1:**
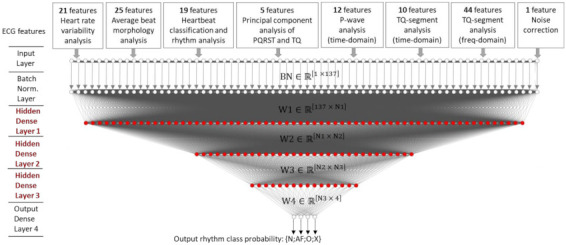
General DenseNet architecture optimized for rhythm classification {N,AF,O,X} with 137 input ECG features, batch normalization layer and configurable dense layers (depth, size): {number dense layers} = [1–4], number nodes@N{1–3} = [0–1024] (red dots). W{1–4} stand for all trainable dense layer weight matrices.

The classifier design considers a multi-layer, shallow, dense neural network (denoted as DenseNet) with general topology, as shown in Figure 1.

The implemented DenseNet includes the following layers:

- Input layer with 137 nodes for fusion of all ECG features in Table 2. Raw feature measurements are retained in original scales and native units without applying initial control or preselection mechanisms.
- Batch normalization (BN) layer, employed as a renowned regularization technique that is known to accelerate training [56]. In our model, BN is applied for standardization of the input feature $\left(x\right)$ by removing the mean and scaling to unit variance $z=\frac{x-\mathrm{mean}}{\mathrm{std}.\mathrm{dev}.}$ for each mini-batch. BN transform layer ${\mathrm{BN}}_{\gamma ,\beta }\left(x\right)\equiv \gamma z+\beta$ computes two trainable parameters (γ,β) for each input feature x.
- A sequence of hidden dense (fully connected) layers for ECG feature fusion and multi-level abstraction of feature maps [57]. Considering a maximum number of three hidden dense layers, the output feature map (y^3^) of the input (x) can be computed as [58]:
  (1) $$y^{3}=f^{3}\left(W3*f^{2}\left(W2*f^{1}\left(W1*x+b1\right)+b2\right)+b3\right)$$
  where $f^{i}{|}_{i=1–3}$ denotes the activation function for each layer (embedded as a rectified linear unit activation ReLU: $f\left(z\right)=z\;\mathrm{when}\;z>0,\;\mathrm{and}\;f\left(z\right)=0\;\mathrm{otherwise}$); $\mathrm{Wi}\in {\mathbb{R}}^{\left[\mathrm{Ni}-1\times \;\mathrm{Ni}\right]}$ stand for the weight matrices of the ith dense layer, with size defined by the number of nodes in the current layer (Ni) and previous layer (Ni-1); $\mathrm{bi}\in {\mathbb{R}}^{\left[1\times \;\mathrm{Ni}\right]}$ stand for the biases of the ith dense layer; (∗) denotes matrix-vector product.
- Output classification dense layer with number of nodes corresponding to the defined four target classes C = {N,AF,O,X} linked to SoftMax activation function (σ). It derives the probability $P_{j}\;$ that the input (x) transferred to the output of the third hidden dense layer above (y^3^) belongs to the jth class (j = 1–4):
  (2) $$P_{j}(x\;\in {\;C}_{j}){=\sigma (y}_{j}^{4})=\frac{\exp\left(y_{\mathrm{Cj}}^{4}\right)}{\sum_{C}\exp\left(y_{C}^{4}\right)},{\;\mathrm{where}\;y}_{j}^{4}=W4_{j}{*y}^{3}+b4_{j}\;$$

In the above equation, the superscript index 4 is linked to the output dense layer (y^4^), following the number of layers in Figure 1. Its matrices of weights and biases are denoted as $W4\in {\mathbb{R}}^{\left[N3\times 4\right]}$ and $b4\in {\mathbb{R}}^{\left[1\times 4\right]}$, respectively, where $\left\{W4_{j},b4_{j}\right\}$ define the weights and biases linked to the jth node of the output layer. The SoftMax normalization ensures that the sum of all components of the output vector P_j_ is 1. The detected class label is taken as maximal argument among all probabilities $\underset{j=1–4}{\mathrm{argmax}}P_{j}(x\;\in {\;C}_{j})$.

We further focus on quantifying the importance of the input feature map to produce high probability for specific class prediction in DenseNet. This would help to derive explanatory contributions of the ECG features for true or false rhythm detections rather than considering predictor variables input to a ‘black box’ network. We adapt the strategy of Connection Weights introduced by Olden and Jackson (2002) [59], which has been proven superior than eight other methods for quantifying variable importance [60], i.e., it provided the largest Gower’s coefficient of similarity between the true ranked importance and estimated ranked importance of input variables on a neural network with 500 Monte Carlo simulations. Based on ecological data, Olden et al. (2004) [60] have shown that Connection Weights approach consistently identifies the correct ranked importance of all predictor variables, whereas the other methods either only identify the first few important variables in the network or no variables at all. Although the former study is based on completely different data and networks, the above idea could be generalized and applied to score the importance of the input ECG features as learnt by the DenseNet classifier in this study.

The original idea of Connection Weights [59] is to quantify the importance of the overall path that a feature ($F_{i}$) reaches from the input to the output node of specific class ($C_{j}$) by summing the product of the weights, connecting the related input neuron through all hidden neurons to the response output neuron:

(3) $$Connection\;Weights\left(F_{i},C_{j}\right)=\overset{N1}{\underset{n1=1}{\sum }}W1_{F_{i},n1}\overset{N2}{\underset{n2=1}{\sum }}W2_{n1,n2}\overset{N3}{\underset{n3=1}{\sum }}W3_{n2,n3}W4_{n3,C_{j}}$$

We note that our DenseNet model includes non-linear ReLU activation of the hidden neurons; therefore, we adapt Equation (3) to sum the weights only if their respective neurons are activated. For this purpose, the Boolean activation matrix $\alpha i\in {\mathbb{R}}^{\left[1\times \;\mathrm{Ni}\right]}$ is introduced in Equation (4) for all neurons in the ith hidden layer, which represent their state: $\left(\alpha =1\right)$ if respective neutron is activated, or $\left(\alpha =0\right)$ if the neuron is deactivated. The activation of the neurons depends on the case-specific input feature map $\alpha i(X_{k}$), as defined for the sample $X_{k}$:

(4) $$\begin{array}{c} Case\;Weights\;Importance\left(X_{k},F_{i},C_{j}\right)=\\ =\overset{N1}{\underset{n1=1}{\sum }}\alpha 1_{n1}\left(X_{k}\right)W1_{F_{i},n1}\overset{N2}{\underset{n2=1}{\sum }}\alpha 2_{n2}\left(X_{k}\right)W2_{n1,n2}\overset{N3}{\underset{n3=1}{\sum }}\alpha 3_{n3}\left(X_{k}\right)W3_{n2,n3}W4_{n3,C_{j}} \end{array}$$

**Figure 2:**
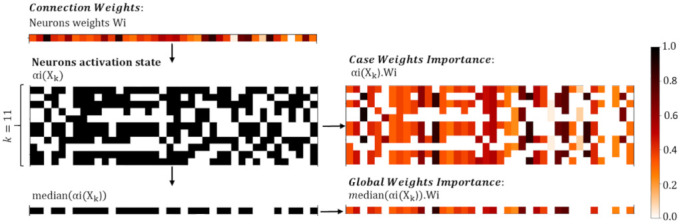
Illustration of the process for computation of Case Weights Importance and Global Weights Importance based on an example neurons weights matrix $\mathrm{Wi}\in {\mathbb{R}}^{\left[1\times 40\right]}$ and neurons activation state matrix $\alpha i\left(X_{k}\right)\in {\mathbb{R}}^{\left[11\times 40\right]}$.

The process for modification of the neuron weight matrices Wi for computation of Case Weights Importance compared to the original Connection Weights is illustrated in Figure 2.

While we are greatly interested on the Global Weights Importance score that is a statistical evaluation of learnt DenseNet kernel weights over the total training dataset, we can eliminate the case specific component $\alpha i(X_{k}$) by taking the median value of the activation matrices computed for the training set, denoted as $\mathrm{median}\left(\alpha i\right)$. The process is illustrated in Figure 2 and defined by the equation:

(5) $$\begin{array}{c} Global\;Weights\;Importance\left(F_{i},C_{j}\right)=\\ =\overset{N1}{\underset{n1=1}{\sum }}\mathrm{median}(\alpha 1_{n1})W1_{F_{i},n1}\overset{N2}{\underset{n2=1}{\sum }}\mathrm{median}(\alpha 2_{n2})W2_{n1,n2}\overset{N3}{\underset{n3=1}{\sum }}\mathrm{median}(\alpha 3_{n3})W3_{n2,n3}W4_{n3,C_{j}} \end{array}$$

We note that the signs of the connection weights W1–W4 are considered, and the result is not scaled, as originally proposed [59,60]. When $Global\;Weights\;Importance\left(F_{i},C_{j}\right)$ is interpreted, the largest absolute values denote the most important features $F_{i}\;$ seen for specific class $C_{j}$, although they could have either positive sign (a high value of the feature contribute to the positive detection of the class) or negative sign (a low value of the feature contribute to the positive detection of the class).

The $Relative\;Feature\;Importance\left(F_{i},C_{j}\right)\;$ is another metric, which is introduced for comprehensive measurement of the importance of a specific feature compared to all others within specific class. The metric is defined to score the best feature normalized to unity for that class:

(6) $$Relative\;Feature\;Importance\left(F_{i},{\;C}_{j}\right)=\frac{Global\;Weights\;Importance\left(F_{i},C_{j}\right)}{\underset{F_{i}}{\max}abs\left(Global\;Weights\;Importance\left(F_{i},C_{j}\right)\right)}\;\;$$

In a case-by-case study, we can use the product of the input receptive field for feature $F_{i}$ with $Case\;Weights\;Importance\left(X_{k},F_{i},C_{j}\right)\;$ for scoring the feature map importance of sample $X_{k}$ passed through the DenseNet kernel matrix towards the prediction of class $C_{j}$:

(7) $$Case\;Feature\;Importance\left(X_{k},F_{i},{\;C}_{j}\right)=\mathrm{BN}\left(X_{k},{\;F}_{i}\right)\;\cdot \;Case\;Weights\;Importance(X_{k},F_{i},C_{j})$$

where the BN layer is used as the corresponding local input receptive field. Case Feature Importance is a sign-dependent score, where the positive sign denotes a feature, which contributes to the positive detection of the class, while the negative sign denotes a feature, which contributes to the negative detection of the class. The features with the largest absolute values of Case Feature Importance are considered to have the most important impact for the detection of specific class.

Equations (1)–(5) are shown for the deepest architecture in this study with four dense layers (DenseNet-4). Nevertheless, they can be easily simplified to fit any shallower topology with less hidden dense layers, as further optimized in the neural architecture search (Section 3.1.1). Any DenseNet-D topology is associated with a specific number of trainable parameters (Params), which is generally linked to the complexity of the model and its time and ability to train, including the problem for overfitting of very large models [61]. In our case, Params is determined by the number of BN layer nodes ${\mathrm{Nodes}}^{\mathrm{BN}}$ (equal to the number of input features), as well as on the number of nodes belonging to D-dense layers:

(8) $$\mathrm{Params}\;\left(\mathrm{DenseNet}-D\right)=2{\mathrm{Nodes}}^{\mathrm{BN}}+\overset{D}{\underset{i=1}{\sum }}{\mathrm{Nodes}}^{i}\left({\mathrm{Nodes}}^{i-1}+1\right)$$

where Nodes^i−1^ for (i = 1) denotes the number of input nodes.

### 2.4. Performance Evaluation and Training

The metrics for reporting DenseNet performance for rhythm classification are further defined, taking into consideration the true positives (TP), false negatives (FN), false positives (FP) and true negatives (TN) in the defined rhythm classes, as follows:

- Global Challenge F1 score computed as an average value of the specific F1-scores for three rhythm classes $R_{x}=\left\{N,\mathrm{AF},O\right\}$, where the Noise class is skipped for averaging due to its small prevalence in 3.3% of the database [22]:
  (9) $$F1\;(\mathrm{Total})=\frac{\sum_{R_{x}=\left\{N,A,O\right\}}F1\left(R_{x}\right)}{3}{,\;\mathrm{where}\;F1(R}_{x})=\frac{2\mathrm{TP}\left(R_{x}\right)}{2\mathrm{TP}\left(R_{x}\right)+\mathrm{FN}\left(R_{x}\right)+\mathrm{FP}\left(R_{x}\right)}$$
- Precision, Recall and Accuracy for all rhythm classes $R_{x}=\left\{N,\mathrm{AF},O,X\right\}$:
  (10) $$\begin{array}{c} \mathrm{Prec}\left(R_{x}\right)=\frac{\mathrm{TP}\left(R_{x}\right)}{\mathrm{TP}\left(R_{x}\right)+\mathrm{FP}\left(R_{x}\right)}\cdot 100\%,\;\mathrm{Recall}\left(R_{x}\right)=\frac{\mathrm{TP}\left(R_{x}\right)}{\mathrm{TP}\left(R_{x}\right)+\mathrm{FN}\left(R_{x}\right)}\cdot 100\%,\\ \;\mathrm{Acc}\left(R_{x}\right)=\frac{\mathrm{TP}\left(R_{x}\right)+\mathrm{TN}\left(R_{x}x\right)}{\mathrm{TP}\left(R_{x}\right)+\mathrm{TN}\left(R_{x}\right)+\mathrm{FP}\left(R_{x}\right)+\mathrm{FN}\left(R_{x}\right)}\cdot 100\% \end{array}$$

For the model design and fitting, we apply ‘Random uniform’ kernel weights initializer and ‘Adam’ optimizer with default learning rate of 0.001 and exponential decay rate for the first and second moment estimates of β1 = 0.9 and β2 = 0.999, respectively. ‘Adam’ optimizes the parameters θ of the network in order to minimize the loss:

(11) $$\theta =\underset{\theta }{\mathrm{argmin}}\frac{1}{N}\overset{N}{\underset{i}{\sum }}\mathrm{Loss}(x_{i},\theta )$$

where x_i_ is the training dataset (or batch size) with a number of N samples, and Loss is computed as a weighted categorical cross entropy due to notable imbalance between output classes:

(12) $$\mathrm{Loss}=-\frac{1}{N}\overset{N}{\underset{i}{\sum }}\overset{C}{\underset{j}{\sum }}{\mathrm{ClassWeights}(C}_{j})\delta_{\mathrm{ij}}{\mathrm{logP}}_{\mathrm{ij}}{(x}_{i}\in {\;C}_{j})$$

where C is the number of classes; ClassWeights is the vector of weights for the classes; $\delta_{\mathrm{ij}}$ is a binary indicator function: $\delta_{\mathrm{ij}}$ = 1 if training sample i has class j, $\delta_{\mathrm{ij}}$ = 0 otherwise; and $P_{\mathrm{ij}}{(x}_{i}\in {\;C}_{j})$ is the predicted probability that sample x_i_ has class j. The value of ClassWeights should respect the condition $\sum_{j}{\mathrm{ClassWeights}(C}_{j})=1$. The weights of different classes represent their imbalance in the training dataset, where the default setting gives a penalty proportional to the class prevalence. However, there could be arbitrary settings for the weights of the classes, so we further investigate to find the optimal setting in Section 3.1.2.

Although not depicted in the general network topology in Figure 1, a drop-out regularization layer is applied after each hidden dense layer to avoid over-fitting and improve generalization during training. Drop-out rate α = 0.3 is adopted as the common setting effectively applied in several previous studies [62,63].

Five independent runs of 5-fold cross-validation are completed for each model. The model with minimal loss over all training epochs is stored in HDF5 file. Early stopping is activated if loss is not improved for >10 epochs. The DenseNet models are implemented in Python using Keras with Tensorflow backend. All experiments are conducted in a workstation PERSY Stinger with Intel CPU Xeon Silver 4214R @ 2.4 GHz (2 processors), 96 GB RAM, NVIDIA RTX A5000-24 GB GPU.

## 3. Results

### 3.1. Neural Network Optimization

#### 3.1.1. Neural Architecture Search

We optimize the two main hyperparameters (HPs) of hidden dense layers that control the architecture of the DenseNet-D network: the number of layers (D) and the number of nodes in hidden layers. We apply a neural architecture grid search of all possible topologies in Figure 1 subjected to the following constraints:

- D = one to four dense layers, including the output dense layer.
- The number of neurons in a given hidden dense layer cannot be larger than the number of neurons in the previous hidden dense layer;
- The number of neurons in a given hidden dense layer can be any in the list {0, 1, 2, 4, 8, 16, 32, 64, 128, 512, 1024}.

**Figure 3:**
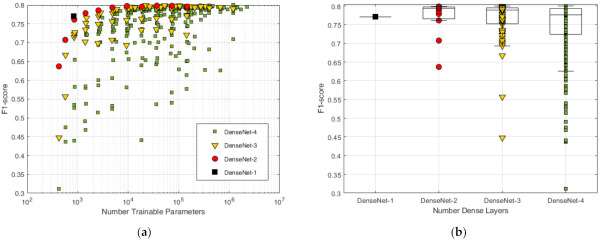
Performance of all DenseNet architectures in the grid search, grouped to the number of dense layers: (**a**) F1 (Total) score observations in respect to the number of trainable parameters; (**b**) Statistical distributions of F1 (Total) score, showing the median value (line), interquartile range 25–75% (boxes), non-outlier range (whiskers) and individual observations (markers).

**Figure 4:**
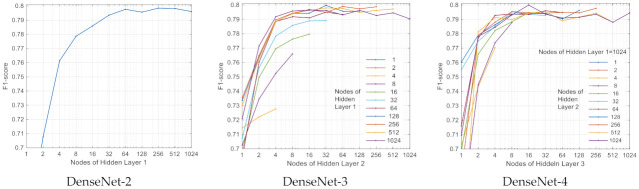
Performance of all NN architectures in the grid search with two (DenseNet-2), three (DenseNet-3) and four (DenseNet-4) dense layers in function of the number of nodes of the last hidden layer.

A number of all possible 364 different DenseNet architectures have been trained, and their cross-validation F1 (Total) score is compared in Figure 3 and Figure 4. The first view in Figure 3a shows the moderate relationship between the performance and the number of trainable parameters (Equation (8)) with a correlation coefficient of 0.48. There is a wide range of trainable parameters [10^4^; 10^6^] seen for top-scored models, although this zone also includes a number of underscored models. Obviously, another HP setting plays a major role in performance. The second view is on the network depth (Figure 3b), where statistical estimation of performance is presented in respect to the number of dense layers. Two topologies (DenseNet-2 and DenseNet-3) present the highest median value of F1-score ≥0.79, although three topologies (DenseNet-2, DenseNet-3 and DenseNet-4) present equally performing best models with maximal F1-score approaching and above 0.799. The third study of performance is related to the number of nodes in hidden layers (Figure 4), where different model architectures are sorted in a comprehensive way. It can be seen that the number of nodes in the last hidden layer strongly affects the performance as soon as they correspond to the feature space into the output classification layer. It could be deduced that the minimal number of last hidden layer nodes should be ≥256 (DenseNet-2), ≥32 (DenseNet-3) and ≥16 (DenseNet-4) in order to reach maximal performance. However, redundant nodes in the last hidden layer are not improving but are more likely to decrease the performance (seen in DenseNet-3 and DenseNet-4).

We further report the topologies of the best performing models with different depths (DenseNet-D@Number neurons in dense layers):

- DenseNet-1@4 (Params = 552): F1-Total = 0.7708;
- DenseNet-2@256-4 (Params = 36,352): F1-Total = 0.7985;
- DenseNet-3@128-32-4 (Params = 21,888): F1-Total = 0.7997;
- DenseNet-4@1024-1024-16-4 (Params = 1,206,336): F1-Total = 0.7998.

Our final choice of the optimal topology model is DenseNet-3@128-32-4 based on its maximal performance and small number of trainable parameters.

#### 3.1.2. Class Weights

This study is focused on optimization of the HP setting related to the ClassWeights, which is the major determinant of the loss function in Equation (12), therefore suggested as a potential performance influencer. A full grid search within the space of reasonable class weights for specific rhythms is applied, introducing W≡ClassWeights:

(13) W(N,AF,O)∈{0, 0.05, 0.1, 0.15,….0.5}, respecting the restriction W(N)+W(AF)+W(O)+W(X)=1

**Figure 5:**
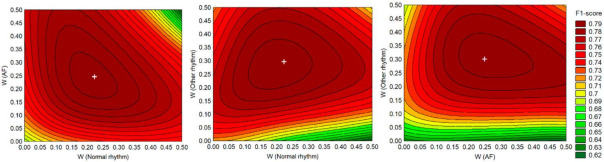
Influence of rhythm class weights W (Normal, AF and Other rhythms) on DenseNet-3@128-32-4 performance. The contour plots of F1-score measured within W grid [0; 0.5] justify the choice of the weights as the average values in the mid-area of maximal performance (+mark): W(Normal) = 0.23, W(AF) = 0.25, W(Other) = 0.3.

A total number of 560 DenseNet-3@128-32-4 models are trained within the pre-defined grid search, and their cross-validation F1 (Total) scores are compared in Figure 5. The class weights considerably influence the performance, showing F1 values in the range 0.62 to 0.79. Our final choice of the optimal class weights is selected as the average values in the mid-area of maximal performance (+mark in Figure 5), corresponding to W(N) = 0.23, W(AF) = 0.25, W(O) = 0.3 and W(X) = 0.22.

#### 3.1.3. Batch Size and Learning Rate

**Figure 6:**
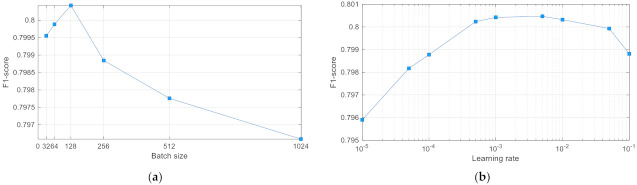
DenseNet-3 performance dependency on batch size (**a**) and learning rate (**b**).

Batch size is the number of signals used in every epoch of NN training that is known to influence convergence and therefore has a huge impact on performance [64]. Six DenseNet-3@128-32-4 models are trained with six respective batch sizes proportional to the power of two within the list {32, 64, 128, 256, 512, 1024}. Their five-fold cross-validation results (Figure 6a) reveal a peak of F1 (Total) score at batch size value equal to 128, which is selected as the optimal HP setting.

Learning rate is related to the convergence rate, and therefore, its value is important toward the possibility of finding the optimal global solution [65]. Nine learning rates covering a wide range from 10^−5^ to 10^−1^, included in the list {0.00001, 0.00005, 0.0001, 0.0005, 0.001, 0.005, 0.01, 0.05, 0.1}, are tested on DenseNet-3@128-32-4 model. The five-fold cross-validation results (Figure 6b) reveal a peak of F1 (Total) score at two intermediate learning rates (0.001 and 0.005). The optimal setting was defined for the learning rate equal to 0.001, which corresponds to the standard default setting of this HP.

#### 3.1.4. Optimal DenseNet Performance

The optimal HP settings derived in Section 3.1.1, Section 3.1.2 and Section 3.1.3 for the DenseNet-3@128-32-4 topology are joined in our ultimate model, which is finally trained for 20 independent runs. The performance of the best models with maximal F1-score by five-fold cross-validation is presented in Table 3 for all rhythm types. The overall performance is: F1 (Total) = 0.802, Acc (Total) = 82.06%, Prec (Total) = 80.34%, Recall (Total) = 82.43%.

### 3.2. Feature Importance

#### 3.2.1. Global Weights Importance

**Figure 7:**
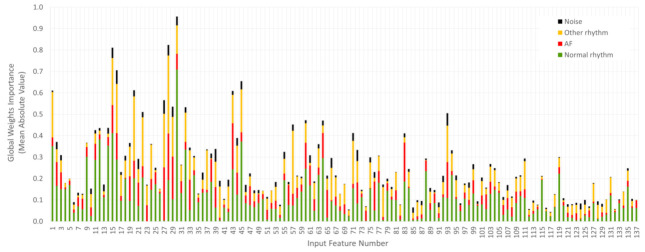
*Global Weights Importance* presented for 137 input features and four rhythm classes. The latter are used to construct stacked barplots, which present the mean absolute values computed by 20 × 5-fold independent runs of DenseNet-3@128-32-4 model. The values are reported without normalization.

The $Global\;Weights\;Importance\left(F_{i},C_{j}\right)$ in Equation (5), which shows the importance of the learnt neural weights, linking a specific input ECG feature $F_{i}\;$ to some of the output rhythm classes $C_{j}$, is investigated for our ultimate DenseNet-3@128-32-4 model reported in Section 3.1.4. In order to assure generalization, the results are statistically evaluated over the trained 100 models from 20 × 5-fold independent runs. The mean absolute value of Global Weights Importance in all runs is computed for i = 137 features and j = 4 classes in Figure 7.

In Figure 7, an uneven distribution of the learnt weights for different classes can be noted to closely relate the class prevalence in the training database (mean value for all features (% from total weights)): 0.11 (46%) for Normal rhythm, 0.069 (28%) for Other rhythm, 0.047 (19%) for AF and 0.017 (7%) for Noise. In order to avoid this class-dependent weights distribution, we further consider the cumulative sum over all classes ($cumGWI=\sum_{C_{j}}Global\;Weights\;Importance$) for ranking the importance of the input ECG features. This value is measured by the height of the stacked bars in Figure 7 that presents certain differences between features. The top-20 ECG features with maximal cumGWI are listed in Table 4, ranging from 0.957 down to 0.412 (not normalized values), corresponding to a normalized feature importance from 1 down to 0.43 (taking the ratio to the maximal cumGWI as a reference). Table 4 discloses additional information for the relative contribution of the top-20 features to each rhythm class by either high value (positive sign) or low value (negative sign) of the estimated Global Weights Importance for that feature. For example, the top-ranked feature ‘P-wave presence’ contributes with a strong positive value (0.709) for Normal rhythms and negative value (−0.074) only for AF class. This trained network information is quite well corresponding to the expert human knowledge on the usual presence of P-waves in the average beat for Normal rhythms and their absence in AF. The second top-ranked feature ‘corBeat(50%)’ presents a negative Global Weights Importance value (−0.050) only for the Noise class, which is in agreement with the human observations for low correlation between heartbeat waveforms disturbed by noise. The next top-ranked HRV features (‘PNN50’, ’PNN50%’, ’SD1/SD2’) present positive values only for AF, being one of the prominent indicators for definitely large RR-interval variability used by experts to recognize this kind of arrhythmia. Another comprehensive feature is ‘Median_TQamp’, which is positive only for the AF class, corresponding to the enhanced f-wave amplitudes typical just for AF arrhythmia. These observations lead us to the conclusion that the NNs self-learn in their hidden weight matrices details for the input features, which are noticeably corresponding to the human knowledge. There might be other details hardly to be catch by the human eye, such as curvatures and their ratios ‘maxc(P)’, ‘maxc(QRS/P)’ or prominently low ‘T-amp’ for AF, which are, however, suggested important by the trained NN weights.

#### 3.2.2. Performance of Reduced Feature Sets Based on Global Weights Importance

**Figure 8:**
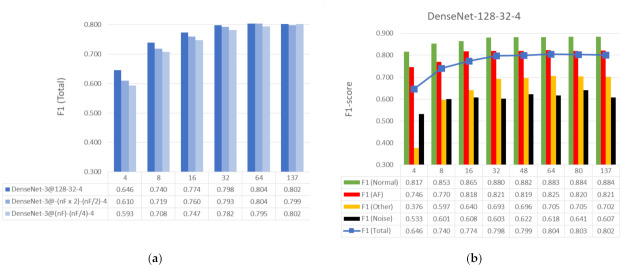
Performance evaluation in case of feature reduction based on *Global Weights Importance* rank for: (**a**) Different architectures of DenseNet; (**b**) DenseNet-3@128-32-4 performance for different rhythms.

The application of Global Weights Importance for feature ranking and reduction of the input feature space is demonstrated in Figure 8. Sets of the top-4, -8, -16, -32 and -64 features, ranked in descending order of their cumGWI in Figure 7, are input to different DenseNet architectures:

1. **DenseNet-3@128-32-4**: The validated architecture for 137 features. We consider that it could be redundant for less features; therefore, some reduced nets are also further defined in (2) and (3);
2. **DenseNet-3@-(nF x 2)-(nF/2)-4**: The first hidden layer is two times the number of input features (nF), and the second hidden layer is four times shorter than the first one. This network can be considered as one two times redundant for input nF;
3. **DenseNet-3@(nF)-(nF/4)-4**: The first hidden layer is equal to the number of input features (nF), and the second hidden layer is four times shorter than the first one. This network can be considered as one shrunk to input nF.

Details on specific architectures and their number of trainable parameters (Equation (8)) are presented in Table 5. Furthermore, the performances of the three DenseNet-3 architectures are compared in Figure 8a. Although the number of trainable parameters can greatly differ, i.e., 72 times (68 vs. 4908 for nF = 4), the F1 (Total) scores of the three DenseNet topologies do not substantially diverge (less than 0.05 points).

Based on the results in Figure 8a, we are further interested on the maximal performance model, which is found for the DenseNet-3@128-32-4 architecture over all feature sets (the darkest blue bars). Furthermore, the detailed performance evaluation of DenseNet-3@128-32-4 over different rhythms is shown in Figure 8b. The reduced set with 64 input features performs equally well as the total of 137 features (F1-Total = 0.804 vs. 0.802). The reduced set with 32 input features can almost reach the performance of 137 features (0.798 vs. 0.802). Small feature sets, including from 4 to 16 input features, present reduced F1-score by 0.156 to 0.028 points, respectively, compared to 137 features. We note maximal performance detection of Normal rhythm, AF, Other rhythm and Noise with at least 16, 16, 32 and 8 features, respectively.

#### 3.2.3. Relative Feature Importance

**Figure 9:**
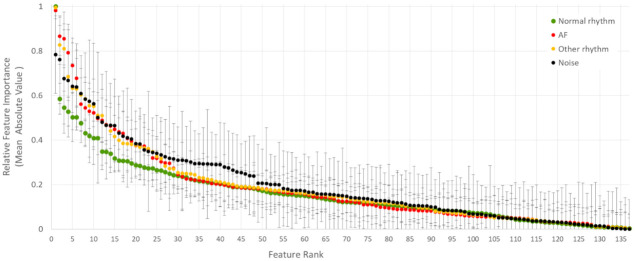
Feature rank given to 137 input features in descending order of their *Relative Feature Importance* to detect specific rhythm class. The dots and whiskers represent the mean absolute values and standard deviations of the *Relative Feature Importance* computed by 20 × 5-fold independent runs of DenseNet-3@128-32-4 model.

Another view on the learnt neural weight matrices is the $Relative\;Feature\;Importance\left(F_{i},C_{j}\right)$ in Equation (6), which can directly rank the importance of a specific input ECG feature $F_{i}\;$ to some of the output rhythm classes $C_{j}$ in a normalized fashion. Using 20 × 5-fold independent runs of DenseNet-3@128-32-4 model, the total number of i = 137 features are ranked according to their mean absolute value (±standard deviation) of the Relative Feature Importance in j = 4 classes, as shown in Figure 9. This representation discards the uneven weights distribution between classes in Figure 7 and normalizes the importance of all features, taking as a reference the maximal unity importance within specific class.

**Figure 10:**
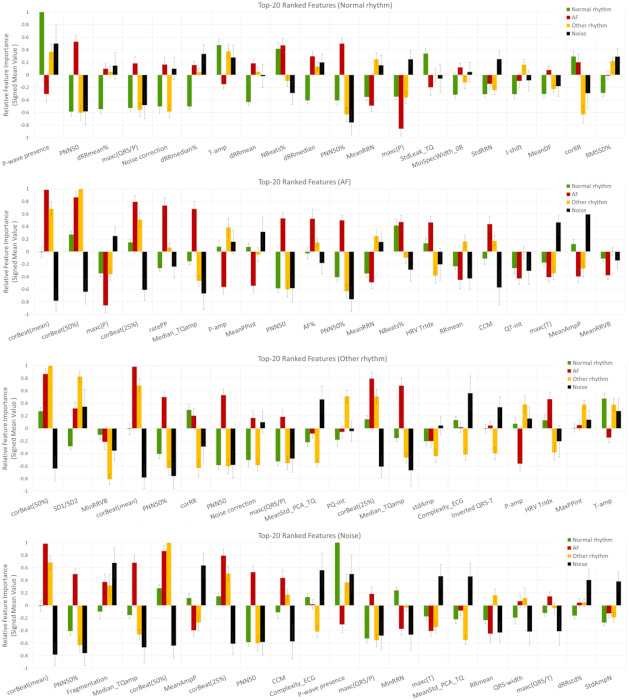
Visual representation of signed *Relative Feature Importance* for the top-20 features ranked in Table 6 to maximally contribute to the detection of each of the four rhythm classes. The bars and whiskers represent the mean values and standard deviations of the *Relative Feature Importance* computed by 20 × 5-fold independent runs of DenseNet-3@128-32-4 model. Signed values of *Relative Feature Importance* are normalized in the range [−1; 1]. They comprehensively indicate the value of the feature that would contribute the positive detection of specific class, i.e., positive sign specifies high feature value and negative sign specifies low feature value.

Additional comprehensive representations of the best feature sets in Table 6 are shown in Figure 10 using signed values for the Relative Feature Importance (normalized in the range [−1; 1]). Here, the importance of the features can be concurrently compared for all classes, showing weather the feature’s pathway from top to bottom of the network is majorly passed through positively or negatively weighted neurons. In the best scenario of well-distinguishable classes, their pathways should have opposite signs. Thus, the feature is concurrently contributing to the positive detection of one class and to the negative detection of the other class. It is also worthy to note that the normalized amplitude is an important measure for the intra-class feature importance, so that the higher the positive or the lower the negative value is, the larger is the contribution for the detection of specific class. Taking the top-ranked features as an example (the left-most features in each subplot of Figure 10), we can reveal that: ‘P-wave presence’ best distinguishes Normal rhythm (maximal positive value) from AF (moderate negative value); ‘corBeat(mean)’ best distinguishes AF (maximal positive value) from Noise (maximal negative value); and ‘corBeat(50%)’ best distinguishes Other rhythm (maximal positive value) from Noise (large negative value). Features, which are plotted by hardly visible bars (Relative Feature Importance close to zero) are weakly contributing to the detection of respective class, such as: ‘dRRmean%’ (AF, Other rhythm, Noise), ‘meanDF’ and ‘RMSSD%’ (AF) and ‘J-shift’ and ‘MinSpecWidth_08’ (AF and Noise), as observed in Figure 9 (top-plot). Overall, the barplot graphs are a comprehensive illustrative tool for highlighting prominent features’ importance and differences between classes as learnt by the NN weight matrices, themselves.

#### 3.2.4. Case Feature Importance

This section is devoted to comprehensive analysis of the input feature map of specific cases, highlighting the most important features that lead to either true or false detection of specific rhythm classes. Such analysis is associated with feature ranking by maximal Case Feature Importance in Equation (7) using the weight matrices of one instance of trained DenseNet-3@128-32-4 model. The decision of the NN is compared to the comprehensive cardiologist’s opinion (author S.N.) on the rhythm diagnostic interpretation. Six examples are further presented, illustrative to six cases of correctly and erroneously detected output classes: Normal rhythm, AF and Other rhythm.

**Figure 11:**
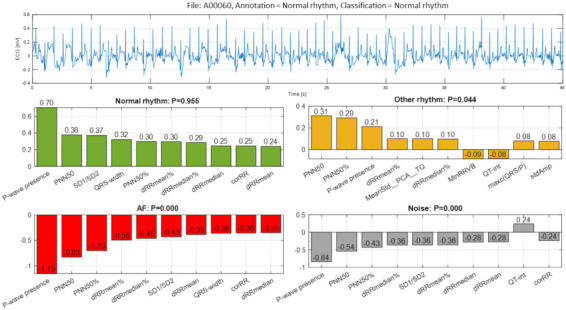
ECG example (file A00060) of correctly classified Normal rhythm. The single-lead ECG strip is representative to sinus rhythm corrupted with baseline and high-frequency artifacts due to poor electrode contact or/and patient’s movements. No discernible repeating P-waves are present, and RR intervals seem ‘irregularly irregular’. The barplots represent the *Case Feature Importance* of the top-10 ranked features, which maximally contribute to the output classification probability (*p*) of four rhythms. The ECG strip is definitively detected as Normal rhythm (P = 0.955) based on HRV features, P-wave presence and QRS-width by DenseNet-3@128-32-4 model.

The first example (Figure 11, file A00060) illustrates a true detection of Normal rhythm with definitive probability P = 0.955 based on the aggregated positive Case Feature Importance contribution of all 10 top-ranked features, including mostly those from HRV analysis (‘PNN50’, ‘SD1/SD2’, ‘PNN50%’, ‘dRRmean%’, ‘dRRmedian%’, ‘dRRmedian’, ‘corRR’, ‘dRRmean’) but also ‘P-wave presence’ and ‘QRS-width’. Meanwhile, the probability for the other output classes is very low (P < 0.05) based on the negative Case Feature Importance contribution of almost all features (except ‘MinRRVB’ and ‘QT-int’). The cardiologist expert opinion on this case is not so definitive, pondering that visual interpretation of this single-lead ECG strip is difficult due to the simultaneous corruption by baseline and high-frequency artifacts and cannot safely exclude AF, particularly in patients with risk factors for this arrhythmia (advanced age, arterial hypertension, ischemic heart disease, etc.). No discernible repeating P-waves are present, and RR intervals seem ‘irregularly irregular’. Such ECG records with artifacts due to poor electrode contact or/and patient’s movements are very common in the real clinical practice during Holter-ECG monitoring and can seriously impede the correct visual classification of the heart rhythm by the human eye.

**Figure 12:**
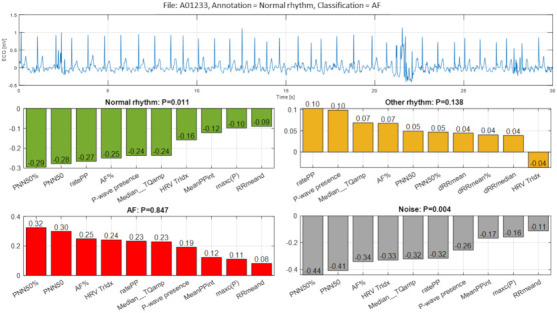
ECG example (file A01233) of erroneously classified Normal rhythm as AF. The single-lead ECG strip is representative to peak artifacts and significant variation of RR intervals in some parts of the record. Visual diagnosis of other dysrhythmias such as atrial flutter and multifocal atrial tachycardia cannot also be safely excluded. The barplots represent the *Case Feature Importance* of the top-10 ranked features, which maximally contribute to the output classification probability (P) of four rhythms. The ECG strip is definitively detected as AF (*p* = 0.847) based on HRV and atrial activity estimation, although Other rhythm (P = 0.138) is also weakly indicated by DenseNet-3@128-32-4 model.

The second example (Figure 12, file A01233) illustrates a false detection of Normal rhythm as AF with explicit probability P = 0.847 due to the aggregated strong positive Case Feature Importance contribution of all 10 top-ranked features based on HRV analysis (‘PNN50%’, ‘PNN50’, ‘HRV Tridx’, ‘dRRmeand’) and atrial activity estimation (‘AF%’, ‘ratePP’, ‘Median_TQamp’, P-wave presence’, ‘MeanPPint’, ‘maxc(P)’). The signal strip is also suggestive to Other rhythm with probability P = 0.138 based on a weak positive Case Feature Importance estimation of the same nine top-ranked features. The labelled class of Normal rhythm is fully rejected (P = 0.011) by all 10 top-ranked features, which present a large negative estimation of Case Feature Importance. The cardiologist expert opinion on this case suggests that the correct visual classification is challenging, and most clinicians would not establish a diagnosis based just on this ECG segment. One suggestive reason to mislead the rhythm as AF could be the peak artifacts and significant variation of RR intervals in some parts of the record. In addition, other dysrhythmias such as atrial flutter and multifocal atrial tachycardia cannot also be safely excluded if this single-lead ECG record is interpreted only visually.

**Figure 13:**
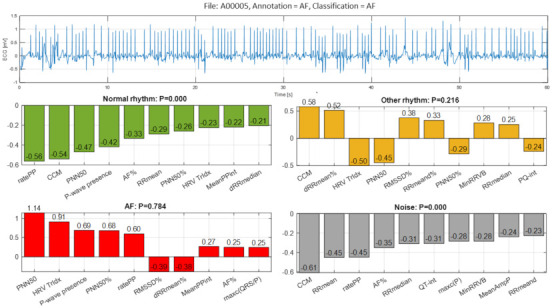
ECG example (file A00005) of correctly classified AF. The single-lead ECG strip is representative of non-sinus, narrow-complex rhythm with high-rate ventricular response and seemingly regular RR intervals in parts. This example presents a certain problem for visual differentiation between high-rate AF, atrial flutter (or alternation of AF and flutter) and AV-nodal re-entrant tachycardia. The barplots represent the *Case Feature Importance* of the top-10 ranked features, which maximally contribute to the output classification probability (P) of four rhythms. The ECG strip is detected as AF with very high probability (P = 0.784) based on HRV and atrial activity estimation, although Other rhythm (P = 0.216) based on six HRV features is also weakly indicated by DenseNet-3@128-32-4 model.

The third example (Figure 13, file A00005) illustrates a true detection of AF with very high probability P = 0.784 due to the aggregated positive Case Feature Importance contribution of eight top-ranked features based on HRV analysis (‘PNN50’, ‘HRV Tridx’, ‘PNN50%’) and atrial activity estimation (‘P-wave presence’, ‘ratePP’, ‘MeanPPint’, ‘AF%’, ‘maxc(QRS/P)’). DenseNet suggests with a smaller probability (P = 0.216) for the presence of Other rhythm based on six top-ranked features representative to HRV but not to atrial or ventricular activity. The main difficulty for clinicians visually classifying the rhythm in Figure 12 is the differentiation between high-rate AF, atrial flutter (or alternation of AF and flutter) and AV-nodal re-entrant tachycardia (AVNRT). Non-sinus, narrow-complex rhythm with high-rate ventricular response and seemingly regular RR intervals at most parts of the record is typical for high-frequency atrial flutter (particularly if flutter is due to the pro-arrhythmic effect of some drugs) and AVNRT. Correct interpretation of this rhythm is important because of the therapeutic approach for the arrhythmias mentioned above is different (AVNRT does not require anti-coagulation in contrast to atrial fibrillation and flutter; different strategy for pharmacological and non-pharmacological restoration of sinus rhythm according to the current guidelines [1]).

**Figure 14:**
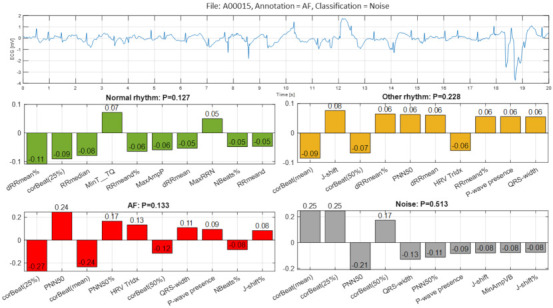
ECG example (file A00015) of erroneously classified AF as Noise. The single-lead ECG strip is representative to significant deflections of the isoelectric line of ‘artifact-type’; not obvious RR irregularity; not clearly seen f-waves of AF. The visual ECG interpretation cannot safely differentiate between sinus rhythm (±supra-ventricular extrasystoles) and AF. The barplots represent the *Case Feature Importance* of the top-10 ranked features, which maximally contribute to the output classification probability (*p*) of four rhythms. The ECG strip is detected as Noise (P = 0.513) due to three top-ranked features representative to large QRS morphology variation. DenseNet-3@128-32-4 model decides with almost equal uncertainty about Other rhythm (P = 0.228), AF (P = 0.133) and Normal rhythm (P = 0.127).

The fourth example (Figure 14, file A00015) illustrates a false detection of AF as Noise with moderate probability P = 0.513 due to the aggregated strong positive Case Feature Importance contribution of three top-ranked features representative to large QRS morphology variation: ‘corBeat(mean)’, ‘corBeat(25%)’ and ‘corBeat(50%)’. The same features, however, contributed strongly negatively to the detection of AF. This seemingly wrong QRS morphology estimation is a consequence from the huge artifacts seen in the ECG record with significant deflections of the isoelectric line of ‘artifact-type’. Furthermore, the decision of DenseNet is hesitating with almost equal uncertainty about Other rhythm (P = 0.228), AF (P = 0.133) and Normal rhythm (P = 0.127). In fact, visual interpretation cannot also safely differentiate between sinus rhythm (±supra-ventricular extrasystoles) and AF. Moreover, RR irregularity is not so obvious, and the f-waves of AF are also not clearly seen. Most clinicians would need additional, non-adjacent ECG leads to classify correctly the underlying rhythm in this ECG strip.

**Figure 15:**
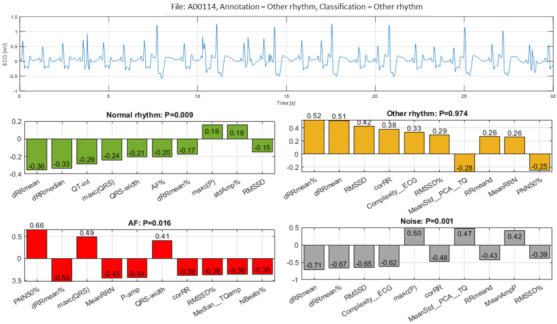
ECG example (file A00114) of correctly classified Other rhythm. The single-lead ECG strip is representative to sinus rhythm with alternating ventricular tri-, quadri- and bi-geminy and a widened baseline QRS as in fixed bundle-branch block. Some of the baseline wide QRS are changed additionally due to artifacts, and they might mislead ventricular ectopy. The barplots represent the *Case Feature Importance* of the top-10 ranked features, which maximally contributed to the output classification probability (P) in four rhythms. The ECG strip is definitively detected as Other rhythm (P = 0.974) by DenseNet-3@128-32-4 model based on HRV and ECG complexity features.

The fifth example (Figure 15, file A00114) illustrates a true detection of Other rhythm with definitive probability P = 0.974 due to the aggregated positive Case Feature Importance contribution of eight top-ranked features: ‘dRRmean%’, ‘dRRmean’, RMSSD’, ‘corRR’, ‘Complexity_ECG’, ‘RMSSD%’, ‘RRmeand’ and ‘MeanRRN’. Those features are representative to HRV and ECG complexity that corresponds to the visual diagnosis of a sinus rhythm with alternating ventricular tri-, quadri- and bi-geminy. The baseline QRS is widened as in fixed bundle-branch block, which, in combination with artifacts in a single-lead ECG, could be challenging for both automatic and visual interpretation: in this case, the grade of ventricular ectopy could be misclassified (bi-, tri-, or quadri-geminy). If heart rate is higher, with no discernible P-waves, the rhythm is frequently mistaken for ventricular tachycardia by many automatic algorithms already in use in the clinical practice. For a clinician (interpreting similar ECG records visually), the main difficulty could be to make a decision if some of the baseline wide QRS are changed additionally due to artifacts or if they are a ventricular ectopy. A fixed bundle-branch block is not infrequent in ECG of patients with advanced heart diseases. However, the same patients have also high risk for ventricular runs that could be ‘obscured’ by the bundle-branch block and artifacts in single-lead ECG records.

**Figure 16:**
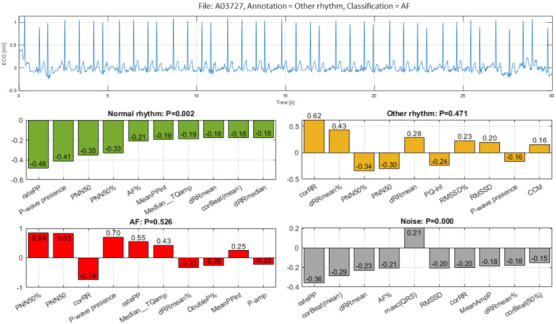
ECG example (file A03727) of erroneously classified Other rhythm as AF. The single-lead ECG strip is representative to sinus rhythm with frequent supra-ventricular extrasystoles and varying RR intervals (due to post-extrasystole compensatory pause); and high-frequency artifacts of the isoelectric line, mimicking f-waves of AF. The barplots represent the *Case Feature Importance* of the top-10 ranked features, which maximally contributed to the output classification probability (P) in four rhythms. The ECG strip is detected as Other rhythm (P = 0.471) and AF (P = 0.526) with almost equal uncertainty by DenseNet-3@128-32-4 model based on HRV and atrial activity features.

The sixth example (Figure 16, file A03727) illustrates a false detection of Other rhythm (P = 0.471) as AF (P = 0.526) with almost equal uncertainty about both rhythms. The erroneous AF classification is mostly due to the strong positive Case Feature Importance contribution of six top-ranked features related to HRV (‘PNN50%’, ‘PNN50’) and atrial activity estimation (‘P-wave presence’, ‘ratePP’, ‘Median_TQamp’, MeanPPint’). Those features are in agreement with the visual interpretation of the underlying rhythm as sinus with frequent supra-ventricular extrasystoles that exhibit varying RR intervals (due to post-extrasystole compensatory pause). Moreover, the high-frequency artifacts of the isoelectric line are mimicking f-waves of AF. These changes could hinder significantly the correct visual interpretation of the ECG by clinicians, and more so in patients with risk factors for AF supra-ventricular extrasystoles, which are common and may co-exist with recurrent episodes of paroxysmal AF.

## 4. Discussion

Rapid advancements of analytical diagnostic systems for point-of-care applications are mainly due to their prompt use at the moment of experiencing symptoms at the site for effective management of severe diseases, even in a personalized manner. It is a point of technological progress that deep learning NNs are replacing the traditional machine learning algorithms used in the everyday practice to support decisions of cardiologists. Nevertheless, this is a long process that requires gaining knowledge on different aspects, one of which is related to investigations towards improving performance, and the second but not less important is related to justification of the decision-making process, itself. Our attention on the decision-making process searches for evidence whether the most important ECG features, which are automatically highlighted by NNs, have meaningful physiological interpretation from the cardiologists’ point of view. This is a kind of clinical justification of the learnt neural network hidden pathways that answers the questions whether they correspond to the traditional ECG markers for specific rhythm diagnosis or suggest novel important ECG features.

In the view of prominent perspectives for fast and accurate point-of-care arrhythmia detection, our study optimizes an advanced artificial NN classifier and ranks the importance of enhanced 137 diagnostic ECG features computed from time and frequency ECG signal representations of short single-lead strips in 2017 Physionet/CinC Challenge database [22]. The automatic detection of arrhythmias with focus on AF is important to the need for prompt and adequate application of different therapeutic strategies for pharmacological and non-pharmacological restoration of sinus rhythm, according to current medical guidelines [1]. Both visual expertise and automated analysis are, however, challenged to identify potentially paroxysmal irregular atrial f-waves activity of AF over very short episodes (9–60 s) in only one ECG lead recorded in non-rigorously controlled conditions. As illustrated (Figure 11, Figure 12, Figure 13, Figure 14, Figure 15 and Figure 16), such conditions are commonly associated with patient movements and poor electrode contact, which are introducing either vast ECG distortions of the baseline, changing the QRS morphology or high-frequency artifacts that are mimicking f-waves. Besides, more non-adjacent ECG leads are missing to confirm the AF diagnosis or to distinguish other supraventricular and ventricular arrhythmias, which might co-exist with recurrent episodes of paroxysmal AF in patients with risk factors (advanced age, arterial hypertension, ischemic heart disease, etc.).

### 4.1. Feature Importance

The presented detailed analysis of the examples in Section 3.2.4 turns attention to the debatable aspects for rhythm interpretation by both human expert and learnt artificial NN classifier. We elucidate the typical black-box NN decision-making process and highlight the input features, which play the most important role for either correct or incorrect automated rhythm classification. A noteworthy contribution of this study is the derivation of the relative to each class feature importance, which clearly distinguishes the separate feature sets accountable for the detection of different rhythms. The results are summarized to a case study by means of the defined Case Feature Importance metric. The latter is greatly appreciated, while the focus is on the comprehensive interpretation of the reasons for a correct or incorrect NN decision based on the case-specific input feature map behaviour (Figure 11, Figure 12, Figure 13, Figure 14, Figure 15 and Figure 16).

The features’ class dependency ranking is also summarized to a global evaluation of the learnt neural weights over the total database. Considering the introduced Relative Feature Importance, which is a normalized metric giving the maximal unity importance of a feature per class (Table 6 and Figure 10), we can distinguish that one feature could differently contribute to the detection of different classes, depending on its pathway from top to bottom of the network through positively or negatively weighted neurons. Outstanding top-ranked feature sets with $\left|Relative\;Feature\;Importance\right|\geq 0.5$ have been identified, which are quite well corresponding to the expert human knowledge on the usual ECG behaviour of different heart rhythms, as follows:

1. AF classification is found to mostly rely on:
  - Positive (‘corBeat(mean)’, ‘corBeat(50%)’, ‘corBeat(25%)’): Estimates high correlation of the morphology of the average beat vs. all beats, which keep normal ventricular depolarization;
  - Positive (‘ratePP’), negative (‘MeanPPint’): Estimates high-rate oscillations of the atrial activity, which are discernible during rapid atrial fibrillation f-waves;
  - Positive (‘Median_TQamp’): Estimates enhanced deflections of the iso-electric TQ intervals, which are discernible during high-amplitude atrial fibrillation f-waves;
  - Negative (‘P-amp’, ‘maxc(P)’): Estimates low-amplitude P-waves in the average beat due to uncoordinated atrial depolarization during AF;
  - Positive (‘PNN50’, ‘PNN50%’, ‘AF%’): Estimates enhanced HRV due to irregularly irregular (i.e., totally irregular) ventricular rate during AF.
2. Normal rhythm classification is found to mostly rely on:
  - Positive (‘P-wave presence’), negative (‘maxc(QRS/P)’): Highlights well-discernible P-wave present in the average beat, considering heart rhythm controlled by the sinus node and each QRS preceded by a normal P-wave without conduction abnormalities;
  - Negative (‘PNN50’, ‘dRRmean%’, ‘dRRmedian%’): Estimates low HRV due to regular ventricular rate, which naturally displays slight beat-to-beat variability controlled by the sympathetic and parasympathetic balance of the autonomic nervous system;
  - Negative (‘Noise correction’): Noise not detected.
3. Other rhythm classification is found to mostly rely on:
  - Positive (‘corBeat(50%)’, ‘corBeat(mean)’, ‘corBeat(25%)’): Estimates high-correlation of the morphology of the average beat vs. 25–50% proportion of all beats, representative to small variance of beat morphology of the sustained rhythm despite that it might include beats with abnormal ventricular depolarization or occasional ectopic beats;
  - Negative (‘PNN50%’, ‘PNN50’): Estimates low HRV determined by the relatively regular ventricular rate of the sustained rhythm;
  - Positive (‘SD1/SD2’) and negative (‘corRR’): Estimates enhanced HRV non-linearity of successive RR-intervals associated with shortened pre-ectopic and prolonged post-ectopic compensatory pause of occasional atrial and ventricular extrasystoles, which are commonly present in Other rhythms;
  - Negative (‘MinRRVB’): Outlines very short minimal RR-intervals associated with tachyarrhythmias or short-coupled ventricular ectopic beats (e.g., R-on-T phenomenon);
  - Negative (‘Noise correction’): Noise not detected;
  - Negative (‘maxc(QRS/P)’): Finds low-slope QRS morphology associated with abnormal ventricular depolarization;
  - Negative (‘MeanStdPCA(TQ)’): Estimates sustained atrial activity during TQ-intervals indicative to regular atrial depolarization preceding QRS;
  - Positive (‘PQ-int’): Finds prolonged PQ-intervals, which are mainly due to slowing of conduction between the atria and ventricles, e.g., discernible in 1st degree AV blocks belonging to the group of Other rhythms.
4. Noise classification is found to mostly rely on:
  - Negative (‘corBeat(mean)’, ‘corBeat(50%)’, ‘corBeat(25%)’): Estimates low correlation of the morphology of the average beat vs. all beats, due to arbitrary noise waveform contamination of most beats.
  - Positive (‘Fragmentation’): Estimates QRS morphology alteration due to prominent noise impact;
  - Negative (‘PNN50%’, ‘PNN50’, ‘CCM’): Estimates low HRV determined by the relatively regular ventricular rate of the sustained rhythm;
  - Positive (‘Complexity_ECG’): Outlines high ECG complexity due to random noise impact on the ECG waveform;
  - Negative (‘Median_TQamp’): Estimates low deflections of the isoelectric TQ intervals in normal or other baseline rhythm with regular atrial depolarization preceding QRS for the most part of signals in the noise group;
  - Positive (‘MeanAmpP’, ‘P-wave presence’): Highlights well-discernible P-wave amplitude and P-wave presence in the average beat, considering a baseline rhythm with regular atrial depolarization preceding QRS.

The highlighted above features have a general importance to the detection of specific rhythms and could be considered in the general setting, where detection of any of the target classes (AF, Normal rhythm, Other rhythm or Noise) is requested outside the framework of the AF detection challenge.

We further investigate the minimal feature set selection process, which should normally merge the features with the greatest importance to the overall group of classes. This feature ranking is based on the Global Weights Importance metric and its cumulative sum over all classes, estimated as cumGWI in Table 4. Among the 20 top-ranked features for all rhythms, we denote definitively the same features as bulleted above for specific classes, though additionally ordered in respect to their importance to the overall database. Although this global feature ranking might depend on the prevalence of rhythms in the specific database, we consider it as a valuable basis for an objective highlight of the features with the greatest importance together for all target classes as deduced from a real-life framework. It is noteworthy to mention the top-contributing features and related analyses to the global NN decision process, as found in this study:

- Average beat morphology analysis with a focus on the P-wave estimation by amplitude (‘P-wave presence’) and curvature (‘maxc(P), ‘maxc(QRS/P)’);
- Average beat morphology analysis with a focus on its correlation to all beat waveforms—‘corBeat(50%)’, ‘corBeat(mean)’, ‘corBeat(25%)’;
- HRV analysis with a focus on the RR–Tachogram (‘PNN50’, ‘PNN50%’), Poincaré Plot (‘SD1/SD2’, ‘corRR’) and dRR–Tachogram (‘dRRmedian%’, ‘dRRmean%’, ‘dRRmedian)’;
- Noise detection based on QRS detector failure—’Noise correction’;
- Average beat morphology analysis with a focus on the PQRST delineation—’T-amp’;
- TQ-segment analysis with a focus on the atrial activity estimation by amplitude (‘Median_TQamp’) and sustainability (‘MeanStd_PCA_TQ’);
- Heartbeat classification and rhythm analysis with a focus on the proportion of the detected normal beats (‘NBeats%’) and related RR intervals (‘MeanRRN’, ‘MinRRVB’).

It is remarkable that NNs have self-learnt this important information, which is covering the basic human knowledge for discerning arrhythmias but is also highlighting specific details hardly to be catch by the human eye. The reduction of the input feature map is of great benefit, which would considerably relieve the pre-processing resources for computation of the input features in part. An overview to the not-selected features shows several laborious analyses, which seem redundant to the ECG diagnosis and could be potentially skipped in future applications, such as: HRV analysis (RR-Histogram), Principal component analysis of PQRST, TQ-segment analysis (complexity and leakage in the time domain) and TQ-segment analysis (frequency domain).

### 4.2. Neural Network Optimization

Another important contribution of this study is the systematic optimization approach applied to the NN classifier with configurable dense neural architecture DenseNet (Figure 1) with results presented in Section 3.1 for the total set of 137 features and Section 3.2.2 for reduced feature sets with 4, 8, 16, 32 and 64 features (selected by maximal cumGWI). In a first step, the optimal HPs of DenseNet with 137 features have been defined by grid search in an extensive range of:

- Number of dense layers (Figure 3): In the scanned range from one to four layers, several models with two, three and four dense layers and trainable parameters in the range [10^4^; 10^6^] are found to reach similar top performances with F1 (Total) > 0.798. Nevertheless, the one with maximal performance and minimal number of trainable parameters (21,888) is found with three dense layers;
- Number of neurons in hidden layers (Figure 4): In the scanned range from 0 to 1024 nodes per layer, the number of nodes in the last hidden layer, corresponding to the feature space into the output classification layer, has been found to be the one that most strongly affects the performance. It could be deduced that the minimal number of nodes in the last hidden layer should be ≥256 (DenseNet-2), ≥32 (DenseNet-3) and ≥16 (DenseNet-4) in order to reach maximal performance. However, redundant nodes in the last hidden layer are not improving but are more likely to decrease the performance (seen in DenseNet-3 and DenseNet-4).
- Class Weights (Figure 5): In the scanned range from 0 to 0.5, we found large deviation of performance F1 (Total) = 0.62–0.79, locating the optimal zone at a mean of: W(N) = 0.23, W(AF) = 0.25, W(O) = 0.3 and W(X) = 0.22. These optimal class weights are quite balanced compared to the prevalence of classes within the database (Table 1): 60% (N), 9% (AF), 28% (O) and 3% (X). This result suggests that careful optimization of the class weights is highly demandable in imbalanced datasets, considering that the common scenarios are using a default penalty proportional to the class prevalence.
- Batch Size (Figure 6a): In the scanned range from 32 to 1024, a noticeable performance maximum for a batch size of 256 is chosen as an optimal value.
- Learning Rate (Figure 6b): In the scanned range from 0.00001 to 0.1, the default learning rate of 0.001 is chosen as an optimal value within the top-performance zone.

As a final result of this grid search process, our final choice of the optimal topology model is DenseNet-3@128-32-4 based on its maximal performance and small number of trainable parameters. It presents best performance of F1 (Total) = 0.802, Acc (Total) = 82.06%, Prec (Total) = 80.34% and Recall (Total) = 82.43%, as deduced from the five-fold cross-validation confusion matrix in Table 3. The detailed rhythm analysis reveals the highest performance metrics (F1-score/Precision/Recall) for Normal rhythms (88.4/86.2/90.7%), followed by AF (82.1/83.1/81.1%), Other rhythms (70.2/73.3/67.4%) and Noise (60.7/68.0/54.8%). The limited performance for Other rhythms could be explained by the diverse content of this group, which includes all abnormal rhythms except AF. Moreover, noisy ECGs have indefinite behaviour and lack of noise level annotation; therefore, the measured features in almost half of the noisy records seem to correctly represent the underlying rhythm instead of the noise, itself.

The second optimization step, which has been focused on the reduction of the input feature set based on cumGWI ranking, presents comparison of nF = 4, 8, 16, 32, 64 and 137 number of features fed to three DenseNet-3 architectures: optimized model (DenseNet-3@128-32-4), model redundant to nF (DenseNet-3@-(nFx2)-(nF/2)-4) and model shrunk to nF (DenseNet-3@(nF)-(nF/4)-4)), as deduced in Section 3.2.2. Figure 8a shows the superiority of DenseNet-3@128-32-4 over all feature sets. Furthermore, the reduced set with 32 and 64 input features performs equally well to the total of 137 features, as shown in Figure 8b. Small input sets, including 4, 8 and 16 features present reduced F1 (Total) score by 0.156, 0.062 and 0.028 points, respectively, compared to 137 features. We note maximal performance for detection of each output class with least features necessary for Noise (8 features), double more for AF and Normal rhythms (16 features) and four times more for Other rhythms (32 features). This observation could be linked to the common difficulties met by experts for visual discerning of various ECG behavioural details, being most definitive for noisy records and most complex for the diversity of ECG rhythm and conduction disorders included in Other rhythms.

### 4.3. Comparative Performance Evaluation

The achieved best performances in this study with full and reduced feature sets (32, 64 and 137 features) are further compared to other published studies. Considering the vast number of references in the Introduction within the framework of the 2017 Physionet/CinC Challenge, in Table 7 we list only studies definitively employing hand-crafted ECG features but not raw ECG data representations that normally might add to the performance. Considering our best F1 (Total) score of about 0.8, we apply an objective threshold selection criterion (0.8 ± 0.01 points) to identify studies with similar achievements. Most of the listed F1 (Total) scores are thus identified to be either comparable (55%, 11/20 studies) or worse F1 (Total) < 0.79 (25%, 5/20 studies), regardless of the number of computed features (from 32 to 260) and applied classifiers (NN, SVM, linear or quadratic discriminant analysis, decision tree, random forest). We distinguish four studies [29,31,33,35] that deserve attention on their methods because they achieve extra performance gain (up to 0.05 points). Although all of them use various decision tree classifiers, we suggest that the performance gain is due to the extracted ECG features but not on the classifiers, themselves. We reveal that Shao et al. (2018) [29] succeeded in achieving F1 (Total) = 0.820 with only 30 features, which are partially covering the diagnostic information in our 137-feature set. We suggest that the more detailed attention on the Poincaré plot geometry is probably beneficial towards better detection of Normal rhythms (0.91 vs. 0.883) and Other rhythms (0.73 vs. 0.705). A similar conclusion could be deduced from Mukherjee et al. (2019) [33], who has used the same Poincaré plot geometry features and has reported quite similar performance as in [29]: F1 (Total) = 0.823, F1 (Normal rhythms)= 0.91. The slight improvement of F1 (Other rhythms) = 0.77 is probably due to the surplus information of 188 ECG features, including sophisticated analyses of adaptive mathematical morphology, complex noise estimation, adaptive noise removal, detection of life-threatening arrhythmias (asystole, extreme bradycardia and tachycardia, ventricular tachycardia and fibrillation), etc. Kropf et al. (2018) with F1 (Total) = 0.830 [31] have reported similar results for Normal and Other rhythms as [29,33], with additional improvement of F1 (AF) = 0.84 vs. 0.825 (this study) by means of a vast amount of 400 time and frequency domain features. We suggest that this improvement could be due to the use of additional view on the morphology of the atrial signal after removing average beats, as well as on meta-level features (unrevealed weighted combination of single features). Teijeiro et al. (2018) [35] have reported the best performance of F1 (Total) = 0.850 using knowledge-based ECG interpretation by Construe abductive algorithm [66] for regular and non-regular rhythm labelling and further computation of 42 high-level features for both individual heartbeats and full record. The remarkable improvement in all rhythms (from 0.035 to 0.07 points) is suggested from expert criteria elucidation and data re-labelling due to noted annotation errors in the training database. Such re-labelling approach seems appropriate for future research to deal with possible database errors.

## 5. Conclusions

The automatic point-of-care detection of different arrhythmias with a focus on AF is challenging task, considering the potentially paroxysmal occurrence of arrhythmias, AF co-existence with other arrhythmias in patients with risk factors and the accidental noise induction that could compromise the diagnostic decision in short-term episodes. Considering the significant burden to patients and healthcare systems globally related to AF complications, the early diagnosis and treatment of this arrhythmia is of crucial importance. The interest to short-term ECG analysis from eased single-lead ECG data is continuously growing. Although automated diagnostic systems based on various machine learning classifiers and an excess of ECG hand-crafted features has been widely studied on test benches, there is a little knowledge about the most important tangible information used in the decision-making process that could be interpreted in least complexity clinical applications and comprehensively linked to the cardiologists’ diagnostic point of view.

The important contributions of this study could be summarized in two aspects of the automated rhythm detection within the PhysioNet CinC Challenge 2017 framework—the optimization and the interpretation of the decision-making process in artificial NNs, which are commonly treated as black-box classifiers. Firstly, the systematic HP grid search approach of a dense NN classifier led us to the optimal configuration (DenseNet-3@128-32-4), which works equally well with 32 to 137 ECG diagnostic features and presents F1-score of about 0.08. DenseNet-3@128-32-4 is capable for rhythm classification even with very limited sets of 8 and 16 input features, giving a tolerable reduction of its maximal performance by about 0.06 and 0.03 points, respectively. The latter would be on high demand in low resource point-of-care rhythm detection systems, which could rely on the computation of only a few ECG characteristics. Therefore, the second contribution of this study is linked to the investigation of a simple method for computation of the feature map importance based on the weights of the activated neurons through the total path from the input to specific output in DenseNet. Thus, a knowledge for both general and relative input map importance to each output rhythm class has been gained for 137 ECG diagnostic features, lighting up the decision-making process and shrinking the input feature space of DenseNet. The top-ranked 20 ECG features with greatest importance to the detection of each rhythm class and the overall of all classes are listed, clearly highlighting that separate feature sets are accountable for the detection of different rhythms. It is remarkable that DenseNet has self-learnt to expand in the weight matrices of its hidden neurons specific details on the ECG behaviour that are closely corresponding to the human knowledge for discerning arrhythmias but also to highlight specific details hardly to be catch by the human eye. The detailed analysis of some challenging examples comprehensively reveals the reasons for either correct or erroneous classification by NN, comparing together the most important features’ contributions for all output classes. The DenseNet decision is compared to a comprehensive cardiologist’s opinion on the rhythm diagnostic interpretation in the context of the ambiguities that are met by both humans and machines.

## Figures and Tables

**Table 1:** Number of recordings in total database and 5 splits for cross-validation. The rhythm distribution within DB spits is preserved equal to the one in total database.

Rhythm Class	Total	DB Splits for 5-Fold Cross Validation					Rhythm Class
Labelling		DB1	DB2	DB3	DB4	DB5	Distribution
Normal sinus rhythm (N)	5076	1016	1015	1015	1015	1015	59.5%
Atrial fibrillation (AF)	758	152	152	152	151	151	8.9%
Other arrhythmia (O)	2415	483	483	483	483	483	28.3%
Noise (X)	279	56	56	56	56	55	3.3%

**Table 2:** List of computed diagnostic ECG features.

Analysis Type	Feature Index	Feature Name	Description
Noise detection	1	Noise correction	Binary value for triggering noise correction technique in case of QRS detection failure. Method described in [28]
HRV analysis (RR–Tachogram)	2–3	RRmean, RRmedian	Mean and median value of all RR intervals (ms) [28,54]
	4–5	RRstd, RRmeand	Standard, mean deviation of all RR intervals (ms) [28,54]
	6–7	RRstd%, RRmeand%	Proportion of Rstd and RRmeand to RRmean (%) [28,54]
	8	RRrat	Ratio of mean-to-median value of RR intervals [28,54]
HRV analysis (dRR–Tachogram)	9–11	dRRmean, dRRstd, dRRmedian	Mean value, standard deviation and median deviation of all RR intervals first differences dRR (ms) [28,54]
	12–14	dRRmean%, dRRstd%, dRRmedian%	Proportion of dRRmean, dRRstd, dRRmedian to RRmean (%) [28,54]
	15–16	PNN50, PNN50%	Proportion of dRR > 50 ms normalized to dRR total sum (min^−1^) and total number (%) [28,54]
	17–18	RMSSD, RMSSD%	Root mean square of successive RR interval differences (ms) and its proportion to RRmean (%) [28,54]
HRV analysis (RR–Histogram)	19	HRV TrIdx	HRV Triangular Index: Total number of RR intervals divided by the number of RR intervals in the modal bin (7.8125 ms) [28,54]
HRV analysis (Poincaré Plot (RR_n_, RR_n−1_))	20	SD1/SD2	Ratio of minor to major semi-axis of the fitted ellipse [28]
	21	CCM	Complex Correlation Measure quantifying the point-to-point (dynamic) variation of the Poincaré plot [28]
	22	corRR	Pearson’s correlation coefficient, representing the goodness of linear fitting of all points in the Poincaré plot [28]
Average beat morphology analysis (Amplitudes)	23	ECG_range	Min-to-max range amplitude of the ECG signal (mV) [28]
	24	stdAmp	Standard deviation of the peak absolute amplitudes of all detected beats (mV) [28]
	25	stdAmp%	Normalized standard amplitude deviation (stdAmp/meanAmp, %), where meanAmp is the mean peak absolute amplitude of all detected beats [28]
	26	rejAmp%	Proportion of rejected beats for synthesis of the average beat with extreme peak absolute amplitudes outside the range (meanAmp ± stdAmp) [28]
Average beat morphology analysis (Cross-correlation)	27 28 29	corBeat(mean), corBeat(50%), corBeat(25%)	Mean value, 25th and 50th percentile of the maximal cross-correlation coefficient of all detected beats against the average beat [28]
Average beat morphology analysis (Delineation of fiducial points)	30	P-wave presence	Binary test for the detected P-wave using criteria in [28]
	31	QRS-amp	Amplitude difference (R-S) (mV) [28]
	32–33	T-amp, P-amp	Absolute amplitude differences from T-peak, P-peak to the isoelectric Q-point [28]
	34–35	J-shift, J-shift%	Absolute amplitude shift of J-point in respect to Q-point (mV) and its normalized value to QRSpp-amp (%) [28]
	36–38	QRS-width, PQ-int, TQ-int	Durations of intervals between fiducial points (ms): QRS-width = (J-Q), PQ-int = Q-Ppeak, TQ-int =Tend-Q [28]
	39	Fragmentation	Binary test for detected inversion of the slope to the left of the R-peak (R-fragm) that could not be physiologically accepted as Q point, satisfying both criteria: - short interval between R-fragm and R-peak (<80 ms) - small amplitude drop at the point of the slope inversion (R-peak − R-fragm < 30% QRS-amp) [28]
	40	Inverted QRS-T	Binary test for detected opposite signs of QRS-peak and T-peak [28]
	41	LBBB	Binary test for detected specific case of inverted QRS and T-wave, satisfying two additional criteria: (QRS-width > 140 ms) and (T-amp > 1/3 ∗ QRS-amp) [28]
Average beat morphology analysis (Curvatures)	42–47	maxc(QRS), maxc(P), maxc(T), maxc(QRS/P), maxc(QRS/T), maxc(T/P)	Maximal curvatures during P, QRS, T waves and their ratios [28]
Heartbeat classification and rhythm analysis	48–51	MeanAmpVB, MinAmpVB, MaxAmpVB, StdAmpVB	Mean, minimal, maximal values and standard deviation of the amplitudes of all ventricular beats VB (mV) [55]
	52–55	MeanAmpN, MinAmpN, MaxAmpN, StdAmpN	Mean, minimal, maximal values and standard deviation of the amplitudes of all detected normal beats (mV) [55]
	56–59	MeanRRVB, MinRRVB, MaxRRVB, StdRRVB	Mean, minimal, maximal values and standard deviation of RR intervals of all VB (ms) [55]
	60–63	MeanRRN, MinRRN, MaxRRN, StdRRN	Mean, minimal, maximal values and standard deviation of the RR intervals between normal beats (ms) [55]
	64	NBeats%	Proportion of the number of normal heartbeats to the total number of beats (%) [55]
	65	AF%	Probability the rhythm to be AF based on assessment of the RR-intervals irregularity (%) [55]
	66	Complexity_ECG	ECG signal complexity [55]
Principal component analysis of PQRST and TQ-intervals	67–70	MeanStd_PCA_PQRST, MinStd_PCA_PQRST, MaxStd_PCA_PQRST, RangeStd_PCA_PQRST	Mean, minimal, maximal values and range of the standard deviation between the amplitude of samples in all PQRST segments and the corresponding samples in the PQRST first PCA vector (mV) [55]
	71	MeanStd_PCA_TQ	Mean deviation between the amplitudes of samples in all TQ segments and the corresponding samples in the TQ first PCA vector (mV) [55]
P-wave analysis (time domain)	72–75	MeanAmpP, MinAmpP, MaxAmpP, StdAmpP	Mean, minimal, maximal values and standard deviation of the P-waves amplitudes (mV) [55]
	76–79	MeanPPint, MinPPint, MaxPPint, StdPPint	Mean, minimal, maximal values and standard deviation of the intervals between consecutive P-waves (ms) [55]
	80–81	MeanPcountRRint, StdPcountRRint	Mean value and standard deviation of the P-waves number in each RR interval [55]
	82	DoubleP%	Proportion of RR intervals with two or more detected P-waves (%) [55]
	83	ratePP	Rate of P-waves (min^−1^) [55]
TQ-segment analysis (time domain)	84	Complexity_TQ	TQ segments complexity [55]
	85–88	MeanLeak_TQ, MinLeak_TQ, MaxLeak_TQ, StdLeak_TQ	Mean, minimal, maximal values and standard deviation of the leakage, calculated for consecutive TQ segments [55]
	89–92	MeanT_TQ, MinT_TQ, MaxT_TQ, StdT_TQ	Mean, minimal, maximal values and standard deviation of the period (T), measured for consecutive TQ segments (ms) [55]
	93	Median_TQamp	Median amplitude of atrial fibrillatory waves in the TQ segment (mV) [28]
TQ-segment analysis (frequency domain)	94–97	MeanDF, MinDF, MaxDF, StdDF	Mean, minimal, maximal values and standard deviation of the dominant frequency of the power spectrum computed over non-overlapping 4 s intervals (Hz) [55]
	98–101	MeanRI, MinRI, MaxRI, StdRI	Mean, minimal, maximal values and standard deviation of the regularity index, which quantifies the sharpness of the dominant peak in the spectra [55]
	102–105	MeanFSNM, MinFSNM, MaxFSNM, StdFSNM	Mean, minimal, maximal values and standard deviation of the first spectral normalized moment [55]
	106–121	MeanSpecWidth_level, MinSpecWidth_level, MaxSpecWidth_level, StdSpecWidth_level level = {_02,_04,_06,_08}	Mean, minimal, maximal values and standard deviation of the spectral width at 4 different levels (0.2, 0.4, 0.6, 0.8) of the normalized maximum power in the range 3–15 Hz [55]
	122–137	MeanSpecArea_level, MinSpecArea_level, MaxSpecArea_level, StdSpecArea_level level = {_02,_04,_06,_08}	Mean, minimal, maximal values and standard deviation of the spectral area enclosed within 4 different levels (0.2, 0.4, 0.6, 0.8) of the normalized maximum power in the range 3–15 Hz [55]

**Table 3:** Average confusion matrix and performance of the optimized DenseNet-3@128-32-4 model based on 5-fold cross-validation.

Rhythm Classes	Predicted Classification				Performance Metrics			
	N	AF	O	X	F1-Score	Prec. (%)	Recall (%)	Acc. (%)
Normal sinus rhythm (N)	4603	14	433	26	0.884	86.2	90.7	85.8
Atrial fibrillation (AF)	16	615	111	16	0.821	83.1	81.1	96.9
Other arrhythmia (O)	657	101	1627	30	0.702	73.3	67.4	83.8
Noise (X)	67	10	49	153	0.607	68.0	54.8	97.7

**Table 4:** List of the top-20 features ranked in Figure 7 by maximal stacked *Global Weights Importance* accumulated over all rhythm classes (*cumGWI*). The reported values present signed mean values of *Global Weights Importance* computed for each class by 20 × 5-fold independent runs of DenseNet-3@128-32-4 model. Normalized values are additionally reported (in brackets): (1) Columns ‘Normal Rhythm’, ‘AF’, ‘Other rhythm’, ‘Noise’: the proportion of *Global Weights Importance* of specific class to the cumulative value for all classes *cumGWI*; (2) Column ‘All rhythms’: the ratio of *cumGWI* of specific feature to the maximal *cumGWI* of the top-ranked feature.

Feature			*Global Weights Importance* (*GWI*) *				
Rank	Input Number	Name	All Rhythms *cumGWI* (Ratio to max(*cumGWI*))	Normal Rhythm Signed *GWI* (% *cumGWI*)	AF Signed *GWI* (% *cumGWI*)	Other Rhythm Signed *GWI* (% *cumGWI*)	Noise Signed *GWI* (% *cumGWI*)
1	30	P-wave presence	0.957 (1.00)	0.709 (74%)	−0.074 (8%)	0.133 (14%)	0.041 (4%)
2	28	corBeat(50%)	0.824 (0.86)	0.193 (23%)	0.217 (26%)	0.364 (44%)	−0.050 (6%)
3	15	PNN50	0.811 (0.85)	−0.412 (51%)	0.131 (16%)	−0.219 (27%)	−0.049 (6%)
4	16	PNN50%	0.706 (0.74)	−0.288 (41%)	0.124 (18%)	−0.230 (33%)	−0.064 (9%)
5	45	maxc(QRS/P)	0.655 (0.68)	−0.372 (57%)	0.043 (7%)	−0.201 (31%)	−0.038 (6%)
6	20	SD1/SD2	0.612 (0.64)	−0.202 (33%)	0.081 (13%)	0.301 (49%)	0.029 (5%)
7	1	Noise correction	0.611 (0.64)	−0.351 (57%)	0.041 (7%)	−0.212 (35%)	0.007 (1%)
8	43	maxc(P)	0.609 (0.64)	−0.244 (40%)	−0.214 (35%)	−0.133 (22%)	0.019 (3%)
9	27	corBeat(mean)	0.566 (0.59)	−0.004 (1%)	0.248 (44%)	0.250 (44%)	−0.063 (11%)
10	29	corBeat(25%)	0.536 (0.56)	0.104 (19%)	0.199 (37%)	0.185 (35%)	−0.048 (9%)
11	32	T-amp	0.533 (0.56)	0.335 (63%)	−0.036 (7%)	0.137 (26%)	0.025 (5%)
12	22	corRR	0.510 (0.53)	0.205 (40%)	0.052 (10%)	−0.228 (45%)	−0.025 (5%)
13	93	Median_TQamp	0.505 (0.53)	−0.109 (22%)	0.168 (33%)	−0.170 (34%)	−0.058 (12%)
14	60	MeanRRN	0.472 (0.49)	−0.246 (52%)	−0.121 (26%)	0.091 (19%)	0.014 (3%)
15	64	NBeats%	0.471 (0.49)	0.295 (63%)	0.117 (25%)	−0.035 (7%)	−0.023 (5%)
16	57	MinRRVB	0.453 (0.47)	−0.074 (16%)	−0.055 (12%)	−0.294 (65%)	−0.030 (7%)
17	14	dRRmedian%	0.436 (0.46)	−0.354 (81%)	0.039 (9%)	0.017 (4%)	0.026 (6%)
18	12	dRRmean%	0.436 (0.46)	−0.382 (88%)	0.024 (6%)	0.018 (4%)	0.012 (3%)
19	11	dRRmedian	0.426 (0.45)	−0.288 (67%)	0.074 (17%)	0.048 (11%)	0.016 (4%)
20	71	MeanStd_PCA_TQ	0.412 (0.43)	−0.155 (38%)	−0.020 (5%)	−0.200 (49%)	0.037 (9%)

* Positive and negative signs respectively indicate high and low feature values contributing for the positive detection of specific rhythm class.

**Table 5:** Number of trainable parameters (Params) of different DenseNet-3 architectures, which are applied to the reduced feature set nF = 4, 8, 16, 32, 64 and the total 137 features.

Number of Input Features (nF)	4	8	16	32	64	137
DenseNet-3@128-32-4 Params	@(128-32-4) 4908	@(128-32-4) 5432	@(128-32-4) 6480	@(128-32-4) 8576	@(128-32-4) 12,768	@(128-32-4) 22,331
DenseNet-3@(nFx2)-(nF/2)-4 Params	@(8-4-4) 104	@(16-4-4) 252	@(32-8-4) 888	@(64-16-4) 3312	@(128-32-4) 12,768	@(256-64-4) 52,443
DenseNet-3@(nF)-(nF/4)-4 Params	@(4-4-4) 68	@(8-4-4) 148	@(16-4-4) 404	@(32-8-4) 1448	@(64-16-4) 5456	@(128-32-4) 22,331

**Table 6:** *Relative Feature Importance* of the top-20 features ranked in Figure 8 to maximally contribute for the detection of a specific rhythm class, reported as mean absolute value ± standard deviation computed over 20 × 5-fold independent runs of DenseNet-3@128-32-4 model.

Rank	Normal Rhythm		AF		Other Rhythm		Noise	
1	P-wave presence	1.0 ± 0.00	corBeat(mean)	0.98 ± 0.04	corBeat(50%)	0.99 ± 0.02	corBeat(mean)	0.78 ± 0.17
2	PNN50	0.59 ± 0.07	corBeat(50%)	0.87 ± 0.09	SD1/SD2	0.83 ± 0.09	PNN50%	0.76 ± 0.20
3	dRRmean%	0.55 ± 0.07	maxc(P)	0.86 ± 0.12	MinRRVB	0.81 ± 0.08	Fragmentation	0.68 ± 0.25
4	Maxc(QRS/P)	0.53 ± 0.07	corBeat(25%)	0.79 ± 0.10	corBeat(mean)	0.68 ± 0.11	Median_TQamp	0.67 ± 0.25
5	Noise correction	0.51 ± 0.10	ratePP	0.73 ± 0.12	PNN50%	0.63 ± 0.11	corBeat(50%)	0.64 ± 0.20
6	dRRmedian%	0.50 ± 0.04	Median_TQamp	0.68 ± 0.13	corRR	0.63 ± 0.14	MeanAmpP	0.64 ± 0.19
7	T-amp	0.48 ± 0.09	P-amp	0.56 ± 0.12	PNN50	0.60 ± 0.12	corBeat(25%)	0.61 ± 0.17
8	dRRmean	0.43 ± 0.06	MeanPPint	0.54 ± 0.11	Noise correction	0.58 ± 0.10	PNN50	0.58 ± 0.21
9	NBeats%	0.42 ± 0.09	PNN50	0.53 ± 0.10	Maxc(QRS/P)	0.55 ± 0.10	CCM	0.57 ± 0.28
10	dRRmedian	0.41 ± 0.05	AF%	0.52 ± 0.15	MeanStdPCA(TQ)	0.55 ± 0.07	Complexity_ECG	0.56 ± 0.27
11	PNN50%	0.41 ± 0.08	PNN50%	0.50 ± 0.08	PQ-int	0.51 ± 0.10	P-wave presence	0.50 ± 0.29
12	MeanRRN	0.35 ± 0.06	MeanRRN	0.49 ± 0.10	corBeat(25%)	0.51 ± 0.12	Maxc(QRS/P)	0.48 ± 0.21
13	maxc(P)	0.35 ± 0.05	NBeats%	0.47 ± 0.11	Median_TQamp	0.47 ± 0.08	MinRRN	0.47 ± 0.24
14	StdLeak_TQ	0.34 ± 0.08	HRV TrIdx	0.46 ± 0.10	stdAmp	0.44 ± 0.11	Maxc(T)	0.47 ± 0.19
15	MinSpecWidth_08	0.32 ± 0.06	RRmean	0.45 ± 0.15	Complexity_ECG	0.42 ± 0.09	MeanStd_PCA_TQ	0.46 ± 0.21
16	StdRRN	0.31 ± 0.05	CCM	0.44 ± 0.12	Inverted QRS-T	0.40 ± 0.10	RRmean	0.43 ± 0.17
17	J-shift	0.31 ± 0.08	QT-int	0.43 ± 0.10	P-amp	0.39 ± 0.14	QRS-width	0.42 ± 0.20
18	MeanDF	0.31 ± 0.05	Maxc(T)	0.41 ± 0.08	HRV TrIdx	0.38 ± 0.12	Maxc(QRS/T)	0.41 ± 0.22
19	corRR	0.30 ± 0.08	MeanAmpP	0.40 ± 0.09	MaxPPint	0.38 ± 0.07	dRRstd%	0.41 ± 0.18
20	RMSSD%	0.29 ± 0.05	MeanRRVB	0.37 ± 0.08	T-amp	0.38 ± 0.11	StdAmpN	0.38 ± 0.15

**Table 7:** Comparative performance evaluation of this study to other published studies based on the F1-score exactly as reported in the original articles. The selected studies are employing only hand-crafted ECG features and not raw ECG data representations that might add to the performance. A few details on the classification methods and number of features (where available) are disclosed.

Study	Method	F1 (N)	F1 (AF)	F1 (O)	F1 (Total)
This study	Dense neural network (DenseNet-3@128-32-4)				
	137 features	0.884	0.821	0.702	0.802 *
	64 features (selected by *Global Weights Importance*)	**0.883**	**0.825**	**0.705**	**0.804** *
	32 features (selected by *Global Weights Importance*)	0.880	0.821	0.693	0.798 *
Athif et al. (2018) [24]	SVM classifier, 47 features	0.879	0.779	0.673	0.777 *
Liu et al. (2018) [25]	SVM classifier, 33 features	0.908	0.786	0.718	0.800 #
Gliner et al. (2018) [26]	SVM classifier, 61 features	0.89	0.80	0.73	0.81 *
Sadr et al. (2018) [27]	Linear discriminant classifier, 122 features	0.87	0.71	0.63	0.737 *
	Quadratic discriminant classifier, 122 features	0.86	0.61	0.53	0.667 *
	Quadratic neural network, 122 features	0.87	0.74	0.66	0.757 *
Christov et al. (2018) [28]	Linear discriminant classifier, 44 features	0.899	0.809	0.697	0.800 #
Shao et al. (2018) [29]	Decision tree classifier, 30 features	0.91	0.82	0.73	0.82 #
Chen et al. (2018) [30]	Decision tree classifier, morphological coefficients and HRV features	0.90	0.78	0.74	0.805 *
Kropf et al. (2018) [31]	Decision tree classifier, 400 features	0.91	0.84	0.75	0.83 #
Rizwan et al. (2018) [32]	Decision tree classifier				
	74 features	0.889	0.781	0.699	0.790 *
	40 features selected with Minimal Redundancy	0.889	0.791	0.702	0.794 *
	40 Minimal Redundancy features + 4 sparse features	0.890	0.785	0.710	0.795 *
Mukherjee et al. (2019) [33]	Decision tree two-layer binary classifier, 188 features	0.91	0.79	0.77	0.823 *
Smisek et al. (2018) [34]	Multi-stage classifier (SVM + decision tree + threshold-based rules), 260 features	0.90	0.81	0.72	0.81 #
Teijeiro et al. (2018) [35]	Multi-stage classifier (Tree gradient boosting model + LSTM), 42 features	0.92	0.86	0.77	0.85 #
Rouhi et al. (2021) [50]	Random Forest classifier				
	52 features (selected by Logistic Regression)	0.896	0.742	0.719	0.786 *
	56 features (selected by Permutation Testing)	0.899	0.740	0.730	0.790 *
	43 features (selected by Random Forest)	0.898	0.751	0.728	0.792 *
	28 features (selected by SHAP technique)	0.900	0.768	0.733	0.800 *

Bolded values present the best performance of this study. # Results reported for the publicly unavailable hidden set of the PhysioNet CinC Challenge 2017 database, as generated upon the Challenge framework (finished January 2018). * Results reported for the publicly available set of the PhysioNet CinC Challenge 2017 database using X-fold cross validation, as used in this study. These results are always disclosed when available in the publication. Otherwise, the results from Note # are disclosed.

## Data Availability

Publicly available datasets were analysed in this study. This data can be found here: [https://archive.physionet.org/physiobank/database/challenge/2017/], accessed on 1 September 2021. The Matlab code for ECG features calculation is distributed along the PhysioNet Computing in Cardiology Challenge 2017 competitors and is available at: [https://physionet.org/content/challenge-2017/1.0.0/sources/ivaylo-christov-204.zip] and [https://physionet.org/content/challenge-2017/1.0.0/sources/irena-jekova-204.zip], accessed on 1 September 2021.
